# Landscape, Water Quality, and Weather Factors Associated With an Increased Likelihood of Foodborne Pathogen Contamination of New York Streams Used to Source Water for Produce Production

**DOI:** 10.3389/fsufs.2019.00124

**Published:** 2020-02-06

**Authors:** Daniel Weller, Alexandra Belias, Hyatt Green, Sherry Roof, Martin Wiedmann

**Affiliations:** 1 Department of Food Science, Cornell University, Ithaca, NY, United States; 2 Department of Biostatistics and Computational Biology, University of Rochester, Rochester, NY, United States; 3 Department of Environmental and Forest Biology, SUNY College of Environmental Science and Forestry, Syracuse, NY, United States

**Keywords:** agricultural water quality, Listeria, Salmonella, geographic information systems, contaminant sources, GFD, microbial source-tracking

## Abstract

There is a need for science-based tools to (i) help manage microbial produce safety hazards associated with preharvest surface water use, and (ii) facilitate comanagement of agroecosystems for competing stakeholder aims. To develop these tools an improved understanding of foodborne pathogen ecology in freshwater systems is needed. The purpose of this study was to identify (i) sources of potential food safety hazards, and (ii) combinations of factors associated with an increased likelihood of pathogen contamination of agricultural water Sixty-eight streams were sampled between April and October 2018 (196 samples). At each sampling event separate 10-L grab samples (GS) were collected and tested for *Listeria*, *Salmonella*, and the *stx* and *eaeA* genes. A 1-L GS was also collected and used for *Escherichia coli* enumeration and detection of four host-associated fecal source-tracking markers (FST). Regression analysis was used to identify individual factors that were significantly associated with pathogen detection. We found that *eaeA-stx* codetection [Odds Ratio (OR) = 4.2; 95% Confidence Interval (CI) = 1.3, 13.4] and *Salmonella* isolation (OR = 1.8; CI = 0.9, 3.5) were strongly associated with detection of ruminant and human FST markers, respectively, while *Listeria* spp. (excluding *Listeria monocytogenes*) was negatively associated with log_10_ *E. coli* levels (OR = 0.50; CI = 0.26, 0.96). *L. monocytogenes* isolation was not associated with the detection of any fecal indicators. This observation supports the current understanding that, unlike enteric pathogens, *Listeria* is not fecally-associated and instead originates from other environmental sources. Separately, conditional inference trees were used to identify scenarios associated with an elevated or reduced risk of pathogen contamination. Interestingly, while the likelihood of isolating *L. monocytogenes* appears to be driven by complex interactions between environmental factors, the likelihood of *Salmonella* isolation and *eaeA-stx* codetection were driven by physicochemical water quality (e.g., dissolved oxygen) and temperature, respectively. Overall, these models identify environmental conditions associated with an enhanced risk of pathogen presence in agricultural water (e.g., rain events were associated with *L. monocytogenes* isolation from samples collected downstream of dairy farms; *P* = 0.002). The information presented here will enable growers to comanage their operations to mitigate the produce safety risks associated with preharvest surface water use.

## INTRODUCTION

Over the last two decades, the occurrence of multiple foodborne disease outbreaks linked to contamination of preharvest produce by wildlife (Cody et al., 1999; Jay et al., 2007; Kangas et al., 2008; Laidler et al., 2013; Kwan et al., 2014) or surface water (e.g., during irrigation; Gelting et al., 2011, 2015; Mody et al., 2011; Lundqvist et al., 2013; FDA, 2019) have highlighted the role of wildlife and surface water as on-farm sources of foodborne pathogens. As part of the traceback investigation during a 2006 *Escherichia coli* outbreak linked to bagged spinach, the outbreak strain was isolated from both feral pig feces and preharvest water from the implicated farm (Jay et al., 2007). Following this outbreak, growers reported increased pressure to adopt practices to prevent wildlife intrusion into produce fields, including through the removal of on-farm, non-crop vegetation (e.g., forest and wetland cover, hedgerows; Beretti and Stuart, 2008; Karp et al., 2015; Baur et al., 2016). Since non-crop vegetation provides key ecosystem services (e.g., erosion prevention, water filtration; Sweeney et al., 2004) its removal can directly affect environmental health and a farm’s economic resiliency. In fact, studies have shown riparian buffers can prevent up to 90% of nutrients in run-off from entering streams (Schultz et al., 2004) and are effective at reducing fecal inputs into streams (Collins and Rutherford, 2004; Ferguson et al., 2007; Wilkes et al., 2013). Non-crop vegetation removal may, therefore, result in impaired water quality. Despite the potential for negative outcomes following non-crop vegetation removal, there is limited data on the impact of upstream landscape structure on the likelihood of detecting foodborne pathogens in preharvest surface water sources. Landscape structure includes patterns of land use within a watershed (e.g., percent of non-crop vegetation in riparian areas) as well as the presence, location, and distance to potential sources of fecal contamination (e.g., livestock operations, wastewater discharge sites). Thus, additional research on the association between upstream landscape structure and foodborne pathogen contamination of preharvest surface water sources is needed to (i) develop effective strategies for comanaging agricultural watersheds for multiple stakeholder aims (e.g., preventing wildlife intrusion, water quality), and (ii) reduce the unintended consequences of on-farm food safety practices (e.g., removal of non-crop vegetation). One aim of the present study therefore is to characterize the association between upstream landscape structure and foodborne pathogen detection.

Recognizing the produce safety concerns surrounding preharvest water use, the US Food and Drug Administration (FDA) proposed preharvest microbial water quality standards as part of the Food Safety Modernization Act (FSMA). The proposed rule states that the *E. coli* concentration in surface water directly applied to preharvest produce must not exceed a geometric mean of 126 CFU/100-mL or a statistical threshold value (90th percentile) of 410 CFU *E. coli*/100-mL (Food Drug Administration, 2015). The geometric mean and statistical threshold value is calculated using 20 water samples collected over a 2–4 year period (Food Drug Administration, 2015). However, interpretation of *E. coli*-based water quality tests is complicated by spatiotemporal variation in the microbial quality of surface water (Goyal et al., 1977; Hipsey et al., 2008; Payment and Locas, 2011; Pandey et al., 2012; Benjamin et al., 2013; Cooley et al., 2014; Weller et al., 2015b, 2020). For example, 71% (15/21), 63% (33/52), and 6% (2/32) of surface water samples collected from the same site in Upstate New York in 2013 (unpublished), 2014 (Weller et al., 2015a), and 2017 (Weller et al., 2020), respectively, were *Listeria monocytogenes*-positive. *E. coli* levels at this site also varied by > 2 log_10_ MPN/100-mL over the course of the 2017 growing season (Weller et al., 2020). Thus, to improve growers’ ability to identify and address on-farm food safety hazards, targeted approaches that account for this variation are needed. Due to the availability of spatial data (e.g., from government databases, Google), analyses that utilize such data can facilitate identification of factors associated with foodborne pathogen detection in agricultural water (Benjamin et al., 2013; Strawn et al., 2013a; Weller et al., 2015a, 2016); these factors can then be used to develop the aforementioned targeted approaches. Past studies have shown that microbial water quality is affected by the ecological context unique to each water source (e.g., upstream landscape) as well as conditions (e.g., weather, physicochemical water quality) at time of water use (e.g., McEgan et al., 2013a; Bradshaw et al., 2016; Weller et al., 2020). It is therefore essential to understand how contamination risks vary in response to weather, physicochemical water quality, and upstream landscape factors as well as interactions between these factors. Thus, the second aim of this study was to use machine-learning approaches robust to correlation between explanatory factors (i.e., conditional inference trees) to identify combinations of environmental factors that were associated with an increased likelihood of foodborne pathogen detection.

Due to the variability in microbial water quality, past studies concluded that the proposed FSMA standard is not effective at identifying food safety risks associated with preharvest surface water use. Specifically, these studies determined that whether a water source meets the proposed standard is largely a function of when the water samples were collected and was not associated with the presence of food safety hazards at the time of water use (Havelaar et al., 2017; Truitt et al., 2018; Weller et al., 2020). Since the relationship between *E. coli* levels and foodborne pathogen presence in agricultural water varied widely within and between past studies (Edberg et al., 2000; Harwood et al., 2005; Wilkes et al., 2009; Payment and Locas, 2011; Benjamin et al., 2013; Pachepsky et al., 2015; Antaki et al., 2016; Weller et al., 2020), concerns have also been raised about the standard’s reliance on generic *E. coli* as an indicator of potential food safety hazards. In fact, a review that compiled the findings of 40 studies with data on *E. coli* levels and pathogen presence in surface water found a significant association between *E. coli* levels and pathogen presence in only 18% of the datasets (Pachepsky et al., 2015). One potential explanation for this variation in the *E. coli*-pathogen relationship is that *E. coli* is an indicator of fecal contamination and not an index organism for foodborne pathogens. This is problematic since foodborne pathogens are not always fecally-associated. For instance, *L. monocytogenes* is a free-living soil microbe, and *E. coli* and *Salmonella* can naturalize in non-host environments [e.g., water (Hendricks, 1967; Goto and Yan, 2011; McEgan et al., 2013a), submerged aquatic vegetation (Byappanahalli et al., 2003; Whitman et al., 2003; Ksoll et al., 2007; SAVs), soil (Ishii et al., 2010; Nautiyal et al., 2010; Goto and Yan, 2011; NandaKafle et al., 2018)]. Another explanation for variation in the *E. coli*-pathogen relationship between studies (Edberg et al., 2000; Harwood et al., 2005; Wilkes et al., 2009; Payment and Locas, 2011; Benjamin et al., 2013; Pachepsky et al., 2015; Antaki et al., 2016) is that *E. coli* is not host-associated, and as such, *E. coli* levels are indicative of all fecal inflows into a waterway. Thus, even when pathogen contamination is of fecal origin, the strength of the *E. coli*-pathogen relationship may be biased by the presence of other fecal inflows that are not contaminated by the target pathogen. Host-associated markers of fecal contamination may offer one way of identifying fecal sources of foodborne pathogens in agricultural water (Green et al., 2019). Moreover, being able to identify and associate pathogen presence with host-associated fecal inputs, will improve our understanding of the food safety hazards associated with human, wildlife, and livestock fecal inputs, and allow for the development of targeted interventions to manage food safety hazards in agricultural water. Thus, the third aim of the present study was to identify potential pathogen sources by characterizing the relationship between foodborne pathogen detection and (i) host-associated fecal indicators, including fecal source tracking (FST) markers for avian (GFD; Green et al., 2012), canine (DG3; Green et al., 2014b), human (HF183; Green et al., 2014a) and ruminant (Rum2Bac; Mieszkin et al., 2010) fecal contamination, and (ii) upstream sources of fecal contamination (e.g., livestock operations, wastewater discharge sites).

## MATERIALS AND METHODS

### Study Design

**FIGURE 1 |:**
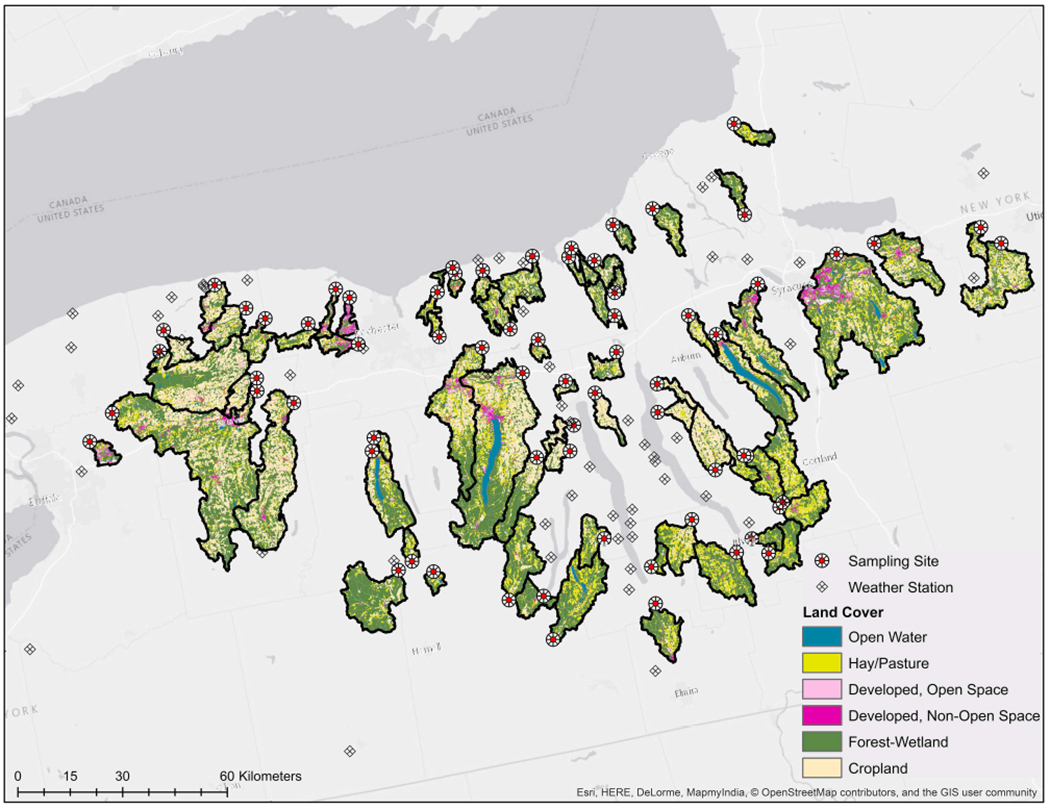
Map showing the study region, including land cover in the sampled watersheds as well as the location of the sampling sites and the NEWA weather stations.

Sixty-eight streams in Upstate New York were sampled two to three times each between April and October 2018 (196 samples total; Figure 1); this timeframe was selected to coincide with the produce growing season in New York. At each sampling, one 10-L grab sample was collected for detection of each set of microbial targets: (i) *Listeria* [*Listeria* spp. (excluding *monocytogenes*) and *L. monocytogenes*], (ii) *Salmonella* and (iii) the *stx* and *eaeA* genes (molecular markers for the potential presence of pathogenic *E. coli*; Hamilton et al., 2010; Melton-Celsa, 2014) as previously described (Weller et al., 2020; 30-L total). A separate 1-L grab sample was also collected to characterize *E. coli* levels. Gloves (Nasco, Fort Atkinson, WI) were changed for each sample collected. All samples were transported on ice and stored at 4°C until samples could be processed. All 10-L grab samples were processed within 18 h of sample collection, while all 1-L grab samples were processed within 6 h of sample collection.

### Grab Sample Processing

The 10-L grab samples were filtered using modified Moore swabs (mMS) as previously described (Sbodio et al., 2013; Weller et al., 2020). After filtration, each mMS was transferred to a separate, sterile Whirl-Pak bag and processed as described below. *E. coli* quantification was performed as previously reported (Weller et al., 2020). Briefly, a 100-mL aliquot of the 1-L grab sample was used for *E. coli* enumeration, which was performed using the Colilert Quanti-Tray 2000 kit (IDEXX, Westbrook, ME) per manufacturer instructions. A second 100-mL aliquot was separately filtered through a 0.45 μm polyethersulfone filter (Whatman, Chicago IL). These filters were then used for detection of FST markers specific to avian, canine, human and ruminant sources as previously described (Table 1). Filters were allowed to thaw prior to adding 29.2 μl of prepared *Caenorhabditis elegans* lysate (Kirtane et al., 2019). The *C. elegans* strain used here contains a *gfp* gene, which can be targeted using the CG4 assay, allowing for (i) estimation of the total amount of DNA recovered from each sample, and (ii) confirmation that qPCR inhibition was absent (Kirtane et al., 2019). Following the addition of the *C. elegans* lysate, the filter and lysate were homogenized using a FastPrep-24-5G (Irvine, CA, MPBio). DNA extraction was then performed using the DNeasy Blood and Tissue Kit (Germantown, MD, Qiagen). Each qPCR reaction consisted of 1X TaqMan Environmental Master Mix (ThermoFisher), DNase and Rnase free water, and assay-specific oligonucleotides (see Table 1). Duplicate reactions were run on a QuantStudio3 or QuantStudio5 (ThermoFisher) under standard cycling conditions: 50°C for 2 min, 95°C for 10 min, and 40 cycles of 95°C for 15 s and 60°C for 1 min. Extraction blanks and three no-template control wells (NTCs) were included in each qPCR run; extraction blanks and NTCs were negative for FST markers in all runs. It is important to note that in the current study, the avian FST assay was modified for hydrolysis probe-based chemistry. The modified GFD assay used the original GFD forward primer [5′-TCG GCT GAG CAC TCT AGG G; Green et al., 2012), a modified GFD reverse primer (5′-GCG TCT CTT TGT ACA TCC CA*T TG*), and a newly-designed ZEN® probe (5′-ACG TCA AGT CAT CAT GGC CCT TAC GC; Coralville, IA, Integrated DNA Technologies). Specificity and sensitivity of the modified GFD assay approximated that of the original SYBR Green-based assay. Specifically, the modified assay was able to correctly identify 86% (13/14) of bird fecal samples (Table S1). Approximately 5% (2/37) of bovine fecal samples were incorrectly identified as being positive for GFD and avian contamination; however, this occurred at very low concentrations per nanogram DNA in the two false-positive samples (Table S1).

### *Listeria* Enrichment and Isolation

*Listeria* enrichment and isolation were performed as previously described (Weller et al., 2015a, 2020). Briefly, 225 mL of buffered *Listeria* enrichment broth (Becton Dickinson, Franklin Lakes, NJ) was added to each Whirl-pak containing a modified Moore swab. Following incubation at 30°C for 4h, *Listeria* selective enrichment supplement (Oxoid, Cambridge, UK) was added to each enrichment. Following incubation at 30°C for a total of 24 h and 48 h, 50 μl of each enrichment were streaked onto *L. monocytogenes* plating medium (LMPM; Biosynth International, Itasca, IL) and Modified Oxford agar (MOX; Becton Dickinson), which were incubated for 48 h at 35 and 30°C, respectively. Up to four presumptive *Listeria* colonies were sub-streaked from MOX to LMPM; the LMPM plates were then incubated at 35°C for 48 h. Up to two presumptive *Listeria* (excluding *L. monocytogenes*) colonies and up to two presumptive *L. monocytogenes* colonies were sub-streaked from LMPM onto brain-heart infusion plates (BHI; Becton Dickinson), which were incubated at 37°C for 24h. For each sample, PCR amplification and sequencing of the partial *sigB* gene (Nightingale et al., 2005; Den Bakker et al., 2010; Bundrant et al., 2011) were used to (i) determine the species of one presumptive *Listeria* (excluding *L. monocytogenes*) isolate, and (ii) confirm one presumptive *L. monocytogenes* isolate as *L. monocytogenes.* The protocol for the *sigB* PCR performed can be found at https://github.com/wellerd2/Laboratory-Protocols. Positive (FSL R3-0001, Roberts and Wiedmann, 2006) and negative controls (uninoculated media) were processed in parallel with all samples.

After processing ~85% of samples, we observed that the prevalence of *Listeria* was substantially lower for this study compared to past NY studies (Strawn et al., 2013a,b; Chapin et al., 2014; Weller et al., 2015a,b). The only methodological difference between our study and past studies was the larger volume of water collected (10-L instead of 250-mL), which necessitated filtration though a mMS instead of a 0.45 um filter. As such, for the last 29 samples collected we filtered 9 L of the 10-L grab sample using the mMs approach and the remaining liter using a 0.45 um filter. We then used McNemar’s χ^2^-square and Cohen’s Kappa to assess the relative ability of paired mMS and 0.45 um filters to detect each *Listeria* species as well as *Listeria* spp. and *Listeria* spp. excluding *L. monocytogenes*. For modeling purposes, a sample was considered positive for a given *Listeria* species if either the mMS or filter were positive for that species.

### *Salmonella* Enrichment and Isolation

Two-hundred twenty-five milliliters of buffered peptone water supplemented with novobiocin to a concentration of 20 mg/L was added to each Whirl-pak containing a modified Moore swab. Following incubation at 35°C for 24 h, *Salmonella* negative samples and presumptive *Salmonella*-positive samples were identified using real-time BAX *Salmonella* assays (Hygiena, Wilmington, DE). BAX negative samples were considered negative for *Salmonella*, while BAX positive samples underwent culture-confirmation for *Salmonella* (Strawn et al., 2013a). Briefly, 1 mL of the enrichment was added to 9 mL of tetrathionate broth (TT; Oxoid) supplemented with 200 μL of I_2_-KI and 100 μL of Brilliant Green. Separately, 0.1 mL of the enrichment was added to 9.9 mL of Rappaport Vassiliadis broth (RV; Acros Organic, Geel, Belgium). After incubating the TT and RV broth in a 42°C shaking water bath for 24h, 50 μL of each broth were streaked onto separate *Salmonella* CHROMagar (DRG International, Springfield, NJ) and xylose lysine deoxycholate agar (XLD; Neogen, Lansing, MI) plates. The plates were incubated at 37 and 35°C, respectively, for 24 h. Up to 12 presumptive *Salmonella* colonies per sample were confirmed as *Salmonella* by PCR amplification of *invA* (Kim et al., 2007) using the protocol for selecting colonies for culture-confirmation described by Weller et al. (2020). The protocol for performing primary and secondary enrichment as well as the BAX Assay can be found in the Supplementary Materials under, *Protocol for Salmonella Detection using the Real-time BAX Salmonella Assay*. The protocol for the *invA* PCR performed here can be found at https://github.com/wellerd2/Laboratory-Protocols. Positive (FSL F6-0826) and negative (uninoculated enrichment media) controls were processed in parallel with all samples.

### *eaeA* and *stx* Detection

A PCR-screen for the *stx* (both *stx1* and *stx2*) and *eaeA* genes was performed using a real-time BAX Shiga-toxin producing *E. coli* (STEC) assay (Hygiena); these genes are considered biomarkers for the potential presence of enteropathogenic *E. coli* (*eaeA*), STEC (*stx*), and enterohemorrhagic *E. coli* (*eaeA* and *stx*). Sample enrichment and processing were performed per manufacturer’s instructions and as previously described (Weller et al., 2020). Briefly, 250 mL of tryptic soy broth supplemented with casamino acids to a final concentration of 10 g/L and with novobiocin to a final concentration of 8 mg/L was added to each Whirl-pak containing a modified Moore swab. The enrichment was then incubated at 41°C for 24h. Following enrichment, the BAX assay was performed per manufacturer’s instructions. The protocol for performing primary enrichment as well as the BAX Assay can be found in the Supplementary Materials under, *Protocol for eaeA-stx Codetection using the Real-time BAX STEC Assay*

### Spatial Data Acquisition and Waterway Enrollment

Hydrological data (www.usgs.gov/core-science-systems/ngp/national-hydrography), USDA Cropscape data on where produce was grown between 2009 and 2017 (nassgeodata.gmu.edu/CropScape/), transportation and infrastructure data (tigerweb.geo.census.gov) and data on the location of public lands (cugir.library.cornell.edu; gis.ny.gov) were obtained to facilitate enrollment of waterways in this study. Watershed delineation and all other spatial analyses were performed using ArcGIS version 10.2 and R version 3.5.3. Watersheds were enrolled by identifying publicly accessible locations (e.g., parks, bridges, Cornell farms) along streams (i) <3.5h from the research laboratory, (ii) with a watershed area of ≥10 km^2^, and (iii) that were <400 m from a field where produce was grown in ≥4 of the years between 2009 and 2017. Flowlines for these watersheds were then converted from linear to point features. Sixty points from non-overlapping watersheds were randomly selected and enrolled as sampling sites in this study. During the course of the study, 11 of the sampling sites had to be replaced (e.g., due to construction, insufficient water). We were able to identify downstream sampling sites that met our enrollment criteria for three of these 11 sites. As a result, we collected three samples from each of these three streams, however, not all samples were collected at the same site; each of these streams is therefore represented by two overlapping watersheds in Figure 1. For the remaining 8 sites, we were unable to identify downstream that met our enrollment criteria. Thus, eight replacement streams were selected using the protocol described above. As a result, 60 streams were sampled three times and eight streams were sampled twice (*N* = 196 samples total). However, because we changed sampling sites for three streams, a total of 71 watersheds are represented in the dataset (3 pairs of overlapping watersheds and 65 non-overlapping watersheds).

To characterize land cover within watersheds, we used inverse-distance weighting (IDW) as described in King et al. (2005). IDW is based on the idea that land cover in areas closer to the sampling site will have a greater impact on water quality than areas farther upstream. The IDW proportion of the total watershed, stream corridor (all area <60 m of the stream channel), and floodplain under each land cover class was calculated (see Table S2 for the list of land covers). Inverse distance weights were calculated using the following distance intervals: 0–100, 100–250, 250–500, 500–1,000, 1,000–2,000, 2,000–5,000, 5,000–10,000, 10,000–20,000, and >20,000 m upstream of the sampling site; all intervals were constrained by either watershed, stream corridor or floodplain boundaries. In addition to characterizing land cover, we also determined if specific landscape features were present upstream of the sampling site (see Table S2 for features considered). If a feature type was present we calculated the upstream flow path distance from the sampling site to the nearest feature. Briefly, flow lines, flow accumulation and flow direction rasters (www.usgs.gov/core-science-systems/ngp/national-hydrography) were used to create flow networks that accounted for overland and in-channel flow using the Hydrology toolset in ArcGIS. The flow networks were imported into R, and the riverdist package (Matt Tyers, 2017) was then used to calculate the flow path distance to the nearest upstream feature for each sampling site and feature type. For features that were also potential sources of fecal contamination (e.g., livestock operations, wastewater discharge sites), the upstream density was also determined. It is important to note that we generated distance and density data for specific types of livestock operations, including dairy farms, poultry farms, and stables, as well as for all livestock operations (i.e., where density includes any livestock operation within the watershed regardless of livestock type). Since septic system data was aggregated at the census tract level (as opposed to being spatially explicit point data like the wastewater discharge sites), upstream septic system density was estimated using the equation below:

$$Density=\frac{\sum \left({\left(\frac{Area{\;of\;Overlap\;Between\;Tract}_{i}\;and\;Watershed}{Area\;of{\;Tract}_{i}}\right)}^{*}No.\;of{\;Septic\;Systems\;in\;Tract}_{i}\right)}{Watershed\;Area}$$

### Weather Data

Weather data were obtained from the NEWA weather station closest to each sample site (Figure 1; http://newa.cornell.edu/). The closest station was identified by drawing Thiessen polygons around all stations in Upstate NY. The average distance between the NEWA stations and the sample sites was 9 km (range = < 1–26 km). If a weather station had a malfunction then data from the next nearest station was used for the time period the malfunction persisted. Average solar radiation and total rainfall for the 0–1, 1–2, 2–3, 3–4, 4–5, 5–10, 10–20, and 20–30 days before sample collection (BSC) was calculated. Due to high Spearman’s correlation between average air temperature 0–1, 1–2, 2–3, 3–4, and 4–5 days BSC, average air temperature for 0–5, 5–10, 10–20, and 20–30 d BSC was calculated. Air and water temperature were also measured in the field at sample collection.

### Statistical Analyses

All analyses were performed in R (version 3.4.2; R Core Team, Vienna, Austria). Correlation between factors was quantified and visualized using the corrplot package (Wei, 2013) as previously described (Weller et al., 2015a). The study reported here tested each sample for multiple microbial targets including key foodborne pathogens *(Salmonella* and *L. monocytogenes)*, pathogen markers (*eaeA* and *stx* genes), and index organisms for key pathogens (non-pathogenic *Listeria*). Separate analyses were performed for each of these microbial targets. Since culture-based methods were used for detection of *Salmonella, L. monocytogenes* and non-pathogenic *Listeria*, and a PCR-screen was used to detect the *eaeA* and *stx* genes, care needs to be taken when comparing results between each microbial targets.

#### Regression Analyses

Generalized linear mixed models (GLMM) were developed to investigate potential relationships between likelihood of detecting each target, and (i) indicators of fecal contamination (e.g., log_10_ *E. coli* concentration, detection of FST markers), (ii) weather and physicochemical water quality factors, and (iii) spatial factors (see Table S2 for a list of all covariates considered). While past studies that investigated potential relationships between pathogens and environmental factors often used Spearman’s correlation to characterize such relationships (e.g., Bradshaw et al., 2016), GLMMs were used here so stream could be included as a random effect. Stream was included as a random effect and week of the year (i.e., no. of weeks since the week that included Jan. 1st) was included as a fixed effect in all GLMMs to account for pseudoreplication in our dataset. The dependent variable in the GLMMs was detection or non-detection of the microbial target. Since this was a hypothesis-generating study, two thresholds were used for interpreting the GLMMs. Specifically, *P* < 0.05 indicated that likelihood of microbial target detection and the factor were significantly associated, while a 0.05 ≥ *P* < 0.10 indicated the presence of a potential relationship that warrants investigation in future studies. When interpreting the results of GLMMs where 0.05 ≤ *P* < 0.10, the magnitude and 95% confidence interval for the change in odds (or odds ratio for categorical explanatory factors) should be considered.

#### Characterizing Spatiotemporal Variation in the Likelihood of Detecting Each Microbial Target

GLMMs were also developed to compare the relative impact of spatial and temporal factors on the likelihood of detecting each microbial target. Using the r.squaredGLMM function in the MuMin package, the marginal (variance accounted for by fixed effects) and conditional (variance accounted for by both fixed and random effects) *R*^2^ were estimated for each model. Temporal fixed effects considered included week of the year, day of the week (e.g., Monday, Tuesday, Wednesday), month (e.g., April, May), and hour of the day. To account for pseudo-replication, stream ID was included as a random effect in all models containing temporal fixed effects. Spatial fixed effects considered were latitude and longitude (to detect linear spatial trends at a scale larger than the watershed-level, e.g., due to N-S land use patterns in New York; Figure 1). Spatial random effects considered included county (to account for non-linear spatial trends at a scale larger than the watershed-level) and stream ID. Table S5 lists all models considered. By comparing the percent variance accounted for in these four models, the relative contribution of space and time to the observed variation in detection of each microbial target could be determined.

#### Conditional Inference Trees (CTrees)

To identify combinations of factors (specific scenarios) associated with an increased or decreased likelihood of detecting each microbial target, CTrees were developed using the mlr and partykit packages. Five-fold cross validation repeated 20 times was used to tune hyperparameters (minbucket and maxdepth) by optimizing the kappa score (Kuhn, 2018). A primary split, competitor split, and two surrogate splits were identified for each CTree node as described by Bradshaw et al. (2016). For CTrees where the outcome was binary and imbalanced (frequency of positive samples was 30% < or 70% >), upsampling was performed as part of tuning (Kuhn, 2018). Due to the use of upsampling, these models may be subject to overfitting (i.e., nodes with higher numbers may be less reliable). To minimize the potential for overfitting we: (i) tuned the maxdepth and minbucket parameters, (ii) limited the upper bound of maxdepth to 10, (iii) limited the lower bound of minbucket to 20, (iv) used a mincriterion of 0.95, and (v) used the Bonferroni multiple comparison correction.

## RESULTS

In the current study, 68 streams were sampled between April and October 2018, and 196 sets of samples were collected (6,078 L total). The area of the sampled watersheds was between 9.6 and 850.0 km^2^ (Mean = 120.8 km^2^; Median = 50.1 km^2^; Table S3). While the proportion of upstream area under any given land cover varied substantially between sites, on average, forest-wetland, cropland, and pasture predominated at all scales of analysis (e.g., whole watershed, stream corridor, floodplain; Table S3). Of the landscape features (e.g., road crossings, culverts) considered here, road crossings were the most common since all streams had at least one upstream bridge. The min. flow path distance from the sampling site to the nearest upstream road crossing ranged between 0.0 and 4.2 km (Mean = 0.5 km). Of the potential sources of fecal contamination considered here, livestock operations and specifically, dairy farms were the most common; dairy farms were present in 63 of the 71 watersheds sampled (note the 71 watersheds correspond to the 68 sampled streams since sampling sites on three of the 68 streams had to be moved). Summary statistics for all factors are reported in Tables S3, S4. Changes in weather and water quality factors over time are shown in Figure S3, while land cover for the sampled watersheds is shown in Figure 1. The only factors that were strongly correlated with time were air and water temperature, which increased from April to August and decreased from August to October (Figures S2, S3).

### Pathogen Prevalence

The presence of potential food safety hazards was determined using culture-based methods to detect *Listeria* (*L. monocytogenes*, and *Listeria* spp. excluding *monocytogenes*) and *Salmonella*, and PCR-based methods to codetect two genes, *stx* and *eaeA*, associated with pathogenic *E. coli* presence; we refer to these collectively within the paper as microbial targets. The most frequently detected targets were the *eaeA* (96%; 190/196 samples) and *stx* (68%; 133/196 samples), which were detected in samples collected from 100% (68/68) and 96% (65/68) of the sampled streams (Table 2), respectively. Temporal factors accounted for between 2% (time of day) and 94% (month) of variance in the likelihood of *eaeA-stx* codetection. Spatial factors accounted for between <1% (latitude) and 10% (longitude) of variance in the likelihood of *eaeA-stx* codetection (Table S5). *Salmonella* was isolated from 40% of samples (79/196). Temporal factors accounted for between 2% (week of the year) and 73% (month) of variance in the likelihood of *Salmonella* isolation, Spatial factors accounted for between 1% (latitude) and 6% (longitude) of variance in the likelihood of *Salmonella* isolation (Table S5). The base model used in the univariate regression analysis accounted for 4% of variance in the likelihood of *Salmonella* isolation (Table S5). *Listeria* spp. (excluding *monocytogenes*) was isolated from 28% (55/196) of samples and 71% (48/68) streams, while *L. monocytogenes* was isolated from 10% (20/196) of samples and 28% (19/68) of streams (Table 2). Temporal factors accounted for between <1% (week of the year) and 4% (month) of variance in the likelihood of *Listeria* spp. (excluding *monocytogenes*) isolation, and for between <1% (time of day) and 8% (month) of variance in the likelihood of *L. monocytogenes* isolation. Spatial factors accounted for between <1% (longitude) and 26% (stream) of variance in the likelihood of *Listeria* spp. isolation, and for between 0% (stream) and 6% (county) of variance in the likelihood of *L. monocytogenes* isolation (Table S5). After collecting 85% of all samples we noted that the prevalence of *Listeria* was substantially lower in this study compared to past NY studies (Strawn et al., 2013a,b; Chapin et al., 2014; Weller et al., 2015a,b). We, therefore, used the last 29 samples collected to compare the ability of paired mMS and 0.45 um filters to detect *Listeria* in 9 L and 1 L of water, respectively. There was significant disagreement in the number of *Listeria* spp. positive samples identified using 0.45 μm filters (18/29) compared to mMs (3/29; *P <* 0.001; Table 3). Based on the Kappa test, the filters were substantially better than the mMS at recovering both *Listeria* spp. (excluding *monocytogenes)* and *L. monocytogenes* (Table 3). For example, the frequency of *L. monocytogenes* detection was 7 times greater using the filters [24%; (7/29)] compared to the mMS [3%; (1/29); Table 3].

### Association Between Indicators of Fecal Contamination and Foodborne Pathogen Detection

Each set of grab samples was tested for five indicators of potential fecal contamination: generic *E. coli* (a non-specific indicator of fecal contamination), and host-associated markers for canine (DG3), avian (GFD), human (HF183), and ruminant (Rum2Bac) fecal contamination. Figure S1 shows how levels of all five fecal indicators changed over the course of the study. *E. coli* was detected in all samples, and *E. coli* levels ranged between 0.3 and 3.4 log_10_ MPN/100-mL (Median = 2.3). Canine, avian, human, and ruminant FST markers were detected in <1% (1/196), 4% (8/196), 25% (49/196), and 17% (34/196) of samples, respectively. The average number of copies/100-mL of the avian, human and ruminant FST markers in samples positive for the respective marker were 1,251 (Min. = 64; Max. = 7,040), 1,643 (Min. = 49; Max. = 320,449), and 1,974 (Min. = 145; Max. = 117,490), respectively (Table S4). The association between likelihood of microbial target detection, and (i) detection and log_10_ concentration of human and ruminant FST markers, and (ii) log_10_ *E. coli* levels were assessed using GLMMs (Table 4). Both *Salmonella* isolation and *eaeA-stx* codetection were positively associated with log_10_ *E. coli* levels (Table 4). For each log_10_ increase in the MPN of *E. coli*/100-mL the odds of isolating *Salmonella* increased 1.8-fold (Odds Ratio [OR] = 1.8; 95% Confidence Interval [CI] = 1.1, 3.1; Table 4). *Salmonella* isolation and *eaeA-stx* codetection were also strongly and positively associated with the detection and log_10_ concentration of human and ruminant FST markers, respectively (Table 4). Specifically, the odds of isolating *Salmonella* approx. doubled (OR = 1.8; 95% CI = 0.91, 3.5) when human FST markers were detected compared to when human FST markers were not detected in the sample. Similarly, the odds of *eaeA-stx* codetection increased by a factor of 4 (OR = 4.2; 95% CI = 1.3, 13.4) when ruminant FST markers were detected in the samples compared to when ruminant FST markers were not detected. While we failed to find evidence of an association between *L. monocytogenes* isolation and any of the fecal indicators considered, the likelihood of isolating *Listeria* spp. (excluding monocytogenes) was negatively associated with *E. coli* levels (OR = 0.50; 95% CI = 0.26,0.96; *P* = 0.036). Due to the low number of DG3 and GFD positive samples, the association between likelihood of microbial target detection, and detection and log_10_ concentration of DG3 and GFD could not be statistically assessed. However, when GFD was present the odds of *Salmonella* isolation and of *eaeA-stx* codetection were both ~1.5 times greater compared to when GFD was not detected [*Salmonella* Odds Ratio [OR] = 1.51 = (4/75)/(4/113); *eaeA-stx* OR = 1.54 = (6/127)/(2/65)]. The sole sample positive for DG3 was also positive for *Listeria* spp. (excluding *monocytogenes*).

### Association Between Environmental Factors and Foodborne Pathogen Detection

GLMMs were used to identify potential relationships between individual weather and water quality factors, and likelihood of microbial target detection; the results of these GLMMs are reported in Table 4 and summarized here. While rainfall 0–1 d BSC and 2–3 d BSC were positively associated with *Salmonella* and *Listeria* spp. (excluding *monocytogenes*) isolation, respectively, rainfall 10–20 d BSC was negatively associated with *Listeria* spp. (excluding *monocytogenes*) isolation. For each one cm increase in rainfall 0–1 d before sample collection, the odds of *Salmonella* isolation increased 2.2-fold (OR = 2.2; 95% CI = 1.31, 3.62), while each one cm increase in rainfall 2–3 d before sample collection the odds of *Listeria* spp. (excluding *monocytogenes*) isolation increased 3.3-fold (OR = 3.3; 95% CI = 1.44, 7.51). Likelihood of *Listeria* spp. (excluding *monocytogenes*) and *Salmonella* isolation were both negatively associated with solar radiation and temperature; *eaeA-stx* codetection was also negatively associated with solar radiation. Additionally, likelihood of *Salmonella* isolation was negatively associated with dissolved oxygen levels and pH, while the likelihood of *Listeria* spp. (excluding *monocytogenes*) was positively associated with both factors. For instance, the odds of *Salmonella* isolation decreased 1.4-fold for each one mg/L increase in dissolved oxygen levels (OR = 0.74; 95% CI = 0.72, 0.89), while the odds of *Listeria* spp. (excluding *monocytogenes*) isolation increased 1.3-fold for each one mg/L increase in dissolved oxygen levels (OR = 1.34; 95% CI = 1.06, 1.69). We also found evidence of a potential strong, positive relationship between *eaeA-stx* codetection and flow rate (OR= 2.90; 95% CI = 0.86, 9.77), and of a negative association between *L. monocytogenes* isolation and log_10_ turbidity levels (OR = 0.32; 95% CI = 0.09,1.08).

Multiple spatial factors were also associated with likelihood of microbial target detection (see Table 5). The likelihood of *eaeA-stx* codetection was positively associated with forest-wetland cover and negatively associated with developed land. For instance, for each 1% increase in the amount of forest-wetland cover in the stream corridor the odds of *eaeA-stx* codetection increased 1.02-fold (OR = 1.02; 95% CI = 1.00, 1.05), while for each 1% increase in developed non-open space in the stream corridor the odds of *eaeA-stx* codetection decreased 1.08-fold (OR = 0.93; 95% CI = 0.86, 1.00). While we did not find significant associations (*P* < 0.05) between land use-factors and *Listeria* spp. (excluding *monocytogenes*), *L. monocytogenes* or *Salmonella* isolation, we did find evidence of potential associations (0.05 < *P* < *0.10*) that warrant future investigation. For example, we found evidence of a negative association between likelihood of *L. monocytogenes* isolation and developed non-open space (OR = 0.96; 95% CI = 0.91, 1.01). Similarly, we found evidence of a positive association between likelihood of *Listeria* spp. (excluding *monocytogenes*) isolation and pasture (OR = 1.03; 95% CI = 95% CI = 1.00, 1.07). In addition to land-use factors, we also found significant associations between upstream features, including hydrological factors (e.g., in-stream waterbodies, ditches, stormwater outfalls) and potential sources of fecal contamination (e.g., livestock operations; Table 5). For example, both the odds of *Salmonella* isolation (OR = 2.04; *P* = 0.042) and the odds of *eaeA-stx* codetection (OR = 2.17; *P* = 0.044) approx. doubled when ditches were present upstream of the sampling site. The odds of *Listeria* spp. (excluding *L. monocytogenes*) isolation was negatively associated with multiple livestock-related factors, including the presence of upstream dairy farms (OR = 0.26; *P* = 0.028) and stables (OR = 0.32; *P* = 0.018) upstream.

### Combinations of Factors Associated With an Increased or Decreased Likelihood of Foodborne Pathogen Detection

**FIGURE 2 |:**
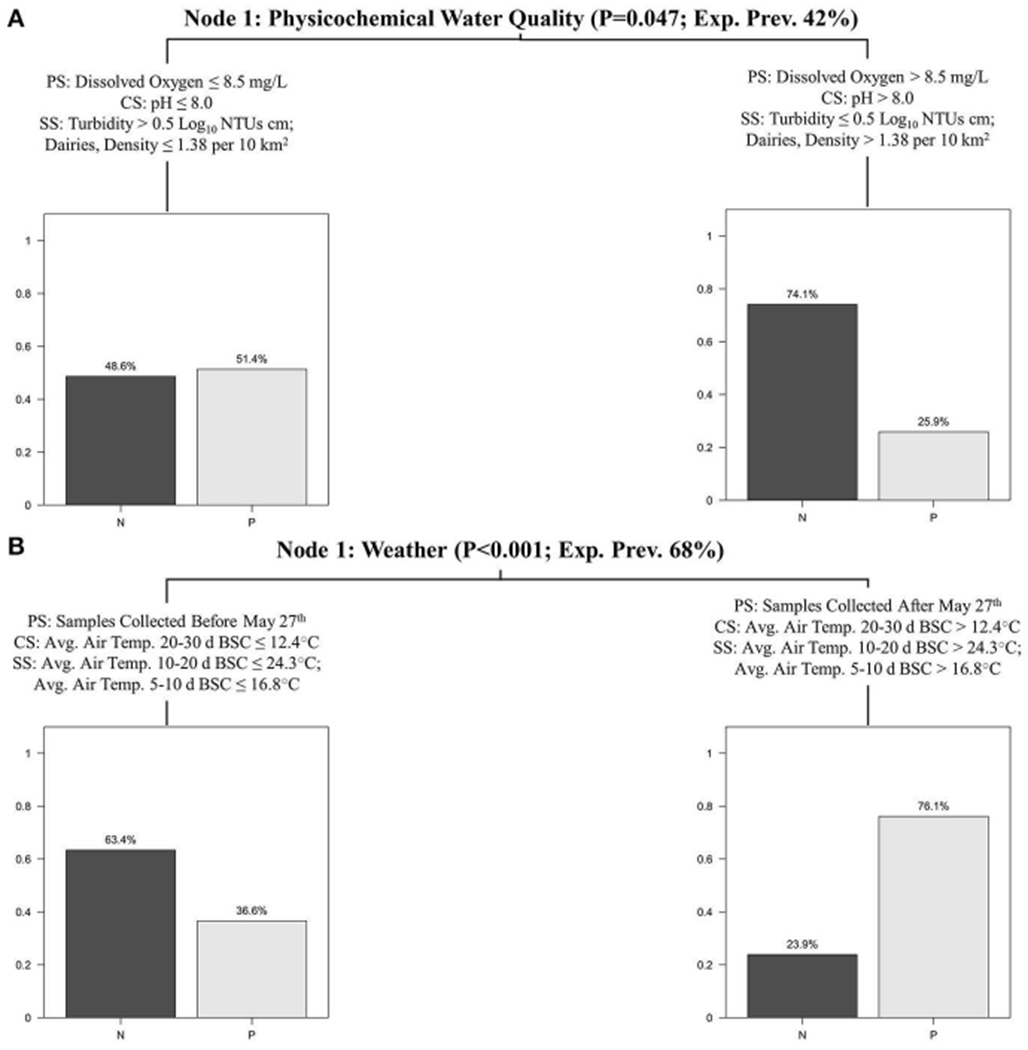
*Salmonella* **(A)**, and *eaeA-stx* **(B)** CTrees showing the primary split (PS), competitor split (CS), and two surrogate splits (SS) for each node. The splits that comprise each node are generalized in the node description (e.g., most splits in node 1A are physicochemical water quality factors). The *p-value* is the *p-value* associated with the primary split for the given node. The expected prevalence in Node 1 was the prevalence of the given microbial target in the study reported here. Bar plots show the exp. prevalence of samples that were negative (N) or positive (P) for the given target(s) in each terminal node.

**FIGURE 3 |:**
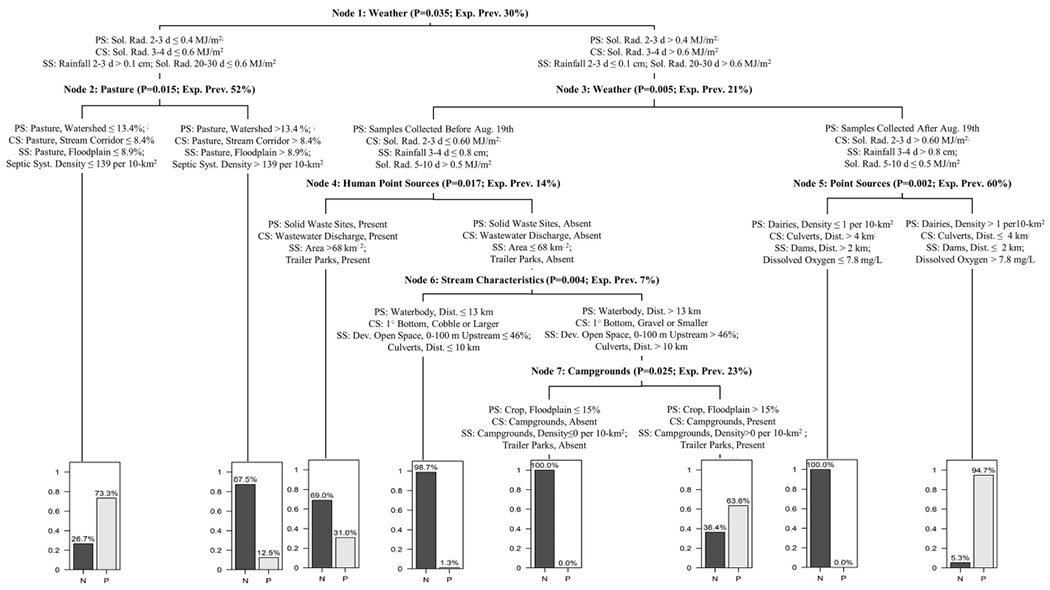
*L. monocytogenes* CTree showing the primary split (PS), competitor split (CS), and two surrogate splits (SS) for each node. The splits that comprise each node are generalized in the node description (e.g., all splits in node 1 are weather factors). Each node description also includes the *P*-value associated with the node’s primary splits, and the expected *L. monocytogenes* prevalence (exp. prev.) based on the node’s parent splits [e.g., exp. prev. in Node 2 for samples collected when avg. solar radiation (sol. rad) 2–3 d BSC was < 0.4 MJ/m^2^ was 52%]. The expected prevalence in Node 1 is greater than that reported in Table 1 since upsampling was used to address the imbalanced nature of the *L. monocytogenes* data when performing CTree analysis. Bar plots show the exp. prevalence of *L. monocytogenes* negative (N) and positive (P) samples in each terminal node. For example, the expected prevalence of *L. monocytogenes* in samples collected on a day when sol. rad 3-4 d BSC was ≤ 0.4 MJ/m^2^ and pasture accounted for > 13.4% of the upstream area was 12.5%.

Conditional inference trees (CTrees) were used to identify and visualize combinations of factors (i.e., specific scenarios) associated with an increased probability of detecting foodborne pathogens in New York agricultural water. CTree results are reported in Figures 2, 3; the CTree where *Listeria* spp. (excluding *L. monocytogenes*) isolation was the outcome did not include any splits and is therefore not reported as a figure. The final CTrees for *Salmonella* isolation and *eaeA-stx* codetection both consisted of a single split (Figure 2). The primary, competitor and first surrogate splits in the *Salmonella* CTree were based on physicochemical water quality at the time of sample collection (Figure 2A). Based on the CTree, the probability of isolating *Salmonella* was highest when dissolved oxygen was below 8.5 mg/L, pH was below 8, or turbidity was above 0.5 log_10_ NTUs. The primary split in the *eaeA-stx* CTree was based on week of the year, while the competitor and surrogate splits were based on air temperature (Figure 2B). Specifically, the probability of *eaeA-stx* codetection was highest between Jun. and Oct. (compared to between Apr. and May), and when air temperature 20–30 d BSC was >12°C, air temperature 10–20 d BSC was >24°C, and air temperature 5–10 BSC was >16.8°C. Since air temperature was strongly correlated with week of the year (Figure S2), the association between *eaeA-stx* codetection and week of the year identified using CTree analysis may be a product of seasonal trends in weather (see Figures S1, S2 and Table S5). The *L. monocytogenes* CTree was more complex than either the *Salmonella* or the *eaeA-stx* CTrees. For instance, the *L. monocytogenes* CTree consisted of four interior and five terminal nodes while the *Salmonella* and *eaeA-stx* CTrees consisted of a single interior node and two terminal nodes. Based on the primary spits, the likelihood of isolating *L. monocytogenes* was greatest when avg. solar radiation 2–3 d before sampling was >0.4 mJ/m^2^, the sample was collected after August 19th, and the upstream density of cattle operations was ≤1 per 10 km^2^. The likelihood of isolating *L. monocytogenes* was lowest when either: (i) avg. solar radiation 2–3 d before sampling was >0.4 mJ/m^2^, the sample was collected after August 19th, and the upstream density of cattle operations was <1 per 10 km^2^, or (ii) avg. solar radiation 2–3 d before sampling was >0.4 mJ/m^2^, the sample was collected before August 19th, and there were no upstream sources of human fecal contamination.

## DISCUSSION

The objectives of this study were to identify (i) potential foodborne pathogen contamination sources using FST markers and geospatial data, and (ii) combinations of spatial, water quality, and weather factors associated with an increased likelihood of detecting foodborne pathogens (*Salmonella* and *L. monocytogenes*), pathogen markers (*eaeA* and *stx* genes), and index organisms for key pathogens (non-pathogenic *Listeria*) in agricultural water samples; we refer to these collectively as microbial targets in the study reported here. As such, this was a hypothesis-generating study, and regression was used to identify factors (i) that were significantly associated with microbial target detection (*P* < 0.05), and (ii) that were not significantly associated with microbial target detection but where a trend that warrants investigation in future studies was present (0.05 < *P* < 0.10). This study is novel due to the diversity of data types used (e.g., weather data, land use data from federal databases, data scraped from Google and government permits, field-collected water quality data), and the computational approaches used to generate these data. For instance, this study calculated flow path distances that account for topography and represent the physical distance a contaminant travels from its source to the sampling site; the Euclidean distance measures used in previous studies (e.g., Strawn et al., 2013a; Weller et al., 2016) do not capture this complexity. However, it is also important to recognize the limitations associated with spatial data. While most of the spatial data is inherently comprehensive (e.g., the wastewater discharge site data includes all sites in NY since the data were generated using NY State permit data) and spatially explicit (e.g., wastewater discharge sites exist as a single point), this is not true for the livestock, campground, or trailer park data. These three data types are based on addresses, and as such, these features, which can cover large land areas (e.g., one campground in the study area is 100 acres), are reduced to single points. This may result in underestimation of feature density, and overestimation of min. flow path distances. For example, dairy farms with addresses outside the watershed but with pastures inside the watershed would be considered absent. As such, the failure to identify significant (*P* < 0.05) associations between livestock operation, trailer park, and campground factors with microbial target detection does not prove a lack of association. Despite this limitation, this study was able to identify potential associations between microbial target detection and these landscape features; as such, these associations should be explored in future studies once more accurate datasets are available. Overall, the integration of diverse data types, as well as the methods used for obtaining said data, is novel and provides a blueprint for how such data and approaches can be used in future studies. This study also illustrates how robust, non-parametric statistical approaches (e.g., conditional inference trees) can be used to investigate how interactions between correlated environmental factors drive foodborne pathogen contamination of preharvest environments.

### Observed *Listeria* Species Prevalence Was Significantly Lower When Modified Moore Swabs, as Opposed to 0.45 μm Filters, Were Used to Process Grab Samples

In this study, we tested all water samples for the presence of *Listeria, Salmonella,* and the *eaeA* and *stx* genes (molecular markers associated with the potential presence of pathogenic *E. coli).* While it is difficult to compare pathogen prevalence between this and past studies (Strawn et al., 2013a,b; Chapin et al., 2014; Weller et al., 2015a,b; Falardeau et al., 2017) due to the larger volume of water collected here (10-L) compared to these studies (between 250 and 532-mL), one would expect a higher prevalence in the current study due to the larger amount of water collected. While the *Salmonella* prevalence observed here was substantially higher than the *Salmonella* prevalence observed in these past studies, it is surprising that the *Listeria* prevalence observed here was substantially lower than the *Listeria* prevalence in these past studies (Strawn et al., 2013a,b; Chapin et al., 2014; Weller et al., 2015a,b). While we isolated *L. monocytogenes* from 10% of samples, studies that sampled the same waterways as the study reported here isolated *L. monocytogenes* from 71% (15/21; unpublished) and 63% [33/52; (Weller et al., 2015a)] of samples collected in 2013 and 2014, respectively. The only methodological difference between our study and these past studies was the larger volume of water collected here, which necessitated filtration though a mMS instead of a 0.45 um filter. Thus, we used the last 29 samples collected to compare *Listeria* recovery using paired mMS and 0.45 um filters. While our analyses are limited by the small sample size, our data indicate that *Listeria* recovery using mMS was significantly lower than *Listeria* recovery using 0.45 um filters, even though 9 times as much water was filtered through the mMS as opposed to 0.45 um filters. In fact, only 14% (2/7) samples that were identified as *L. monocytogenes-positive* using filters were also identified as *L. monocytogenes-positive* using mMS. Since mMS work by capturing large particles (e.g., sediment) to which bacteria are attached, one explanation for the lower than expected *Listeria* prevalence in our study may be differences in attachment mechanisms between *Listeria* and the enteric bacteria species used to validate the mMS approach (Bisha et al., 2011, 2014; McEgan et al., 2013b; Sbodio et al., 2013; Zhu et al., 2019). Indeed, differences in bacterial surface structures, hydrophobicity, surface charge, cell size, and cell sphericity have been shown to affect bacterial attachment to sediment and other surfaces (Faille et al., 2002; Ukuku and Fett, 2002; Vorst et al., 2004; Walker et al., 2005; Silva et al., 2008; Wan Norhana et al., 2009; Liao et al., 2015). One study found a strong correlation between cell surface hydrophobicity and the strength of *Listeria*, *Salmonella,* and *E. coli* attachment to cantaloupe rinds, with *Salmonella* and *E. coli* attaching more strongly to the rind than *Listeria*. Given our findings, follow-up studies are needed to (i) determine why *Listeria* recovery is lower using mMS compared to 0.45 um filters, and (ii) how the mMS approach can be adapted to facilitate *Listeria* recovery. Such studies are essential if mMS are to be incorporated into water testing programs as previously suggested (Bisha et al., 2014). Due to the use of mMS in the present study, the results of analyses where the likelihood of *Listeria* isolation was the outcome need to be interpreted in the context of the sampled population. Thus, factors identified as significant here should be considered associated with *Listeria* isolation from mMS as opposed to *Listeria* isolation from water samples.

### *Salmonella* Isolation and *eaeA-stx* Codetection Were Associated With Human and Ruminant Fecal Contamination, Respectively

In the present study, we found evidence of a strong, positive association between ruminant FST markers (Rum2Bac) and *eaeA-stx* codetection, and between human FST markers (HF183) and *Salmonella* isolation. In general, these findings are consistent with past studies that found strong associations between ruminant fecal contamination and detection of pathogenic *E. coli* markers (Walters et al., 2007; Petit et al., 2017), and between human fecal contamination and *Salmonella* detection (Marti et al., 2013; Liang et al., 2015; Stea et al., 2015). For example, Stea et al. (2015) found that the odds of detecting molecular makers for *Salmonella* in Nova Scotia surface water samples was 2.2 times greater when human FST markers were present as opposed to when human FST markers were not detected. Bradshaw et al. (2016) reported that a model containing log_10_ ruminant FST marker (Rum2Bac) concentration and water temperature was able to identify 100% of *stx*-positive water samples collected in Georgia, USA. Bradshaw et al. (2016) also reported that the odds of *stx* detection increased by a factor of 2 for each log_10_ increase in ruminant FST marker concentration, which is similar to the odds ratio calculated here (OR = 1.6; 95% CI = 1.1, 2.4). The identification of an association between host-associated FST markers, and *Salmonella* and *eaeA-stx* codetection is also consistent with the associations between spatial factors and microbial target detection identified here and in past studies (Sassoubre et al., 2011; Walters et al., 2011; Wilkes et al., 2011; Bradshaw et al., 2016). For instance, Wilkes et al. (2011) identified a positive association between pasture being present 0–5 km upstream of a sampling site, and an increased likelihood of isolating *E. coli* O157:H7 from water samples collected in Ontario, Canada. Interestingly, in the present study, we did not find associations between upstream agricultural land and *eaeA-stx* codetection but did find associations between *eaeA-stx* codetection and forest-wetland cover. The lack of an association between upstream agricultural land use and *eaeA-stx* codetection as well as the identification of an association between forest-wetland cover and *eaeA-stx* codetection may indicate that the ruminant fecal contamination detected in the present study is of cervid as opposed to bovine origin. This is supported by the fact that (i) multiple *E. coli* outbreaks have been attributed to deer intrusion into recreational and farm environments (Cody et al., 1999; Feldman et al., 2002; Laidler et al., 2013), and (ii) past studies have isolated pathogenic *E. coli* from deer feces (Rice et al., 1995; Dunn et al., 2004; Díaz et al., 2011). Despite the potential association between deer fecal contamination and *eaeA-stx* detection in agricultural water samples, the authors are not recommending the removal or alteration of upstream habitat to reduce deer populations. In fact, based on other associations identified here, conversion of forest-wetland cover to developed or agricultural land uses would increase the likelihood of *Salmonella* or *L. monocytogenes* being present in the waterway. This highlights the potential for unintended consequences (increased risk of *Salmonella* or *L. monocytogenes* contamination) when food safety management practices (removal of forest-wetland cover) are implemented with a single aim in mind (such as reducing contamination by pathogenic *E. coli*).

*Salmonella* isolation was positively associated with both human fecal contamination and proxies for increased human presence in upstream areas [proximity to campgrounds, developed open space (e.g., parks)], which is consistent with the existing literature (e.g., Christensen et al., 1978; Varness et al., 1978; Dasher et al., 1981; Hendry and Leggatt, 1982; Flack et al., 1988; Sassoubre et al., 2011; Walters et al., 2011; Vereen et al., 2013). Previous research has shown that increases in recreational activities (e.g., camping, hiking, swimming) can affect the microbial quality of downstream surface water sources (Christensen et al., 1978; Varness et al., 1978; Dasher et al., 1981; Hendry and Leggatt, 1982; Flack et al., 1988), which may explain the association between proximity to campgrounds and *Salmonella* isolation reported here. Interestingly, we also found a positive association between proximity to poultry operations and *Salmonella* isolation; although too few samples tested positive for the avian FST marker to perform statistical analyses, the odds of detecting *Salmonella* when the avian FST marker was present was 1.5 times greater than when the marker was not detected. Overall, the association between avian fecal sources and *Salmonella* isolation is consistent with previous studies (e.g., Vereen et al., 2013; Hernandez et al., 2016; Steele et al., 2018) as well as the fact that *Salmonella* is a well-known contaminant of poultry production systems (Louis et al., 1988; Rodrigue et al., 1990; Behravesh et al., 2014). In a survey of *Salmonella* prevalence in the Satilla River Basin, Vereen et al. (2013) found that sources of poultry and human fecal contamination were positively associated with *Salmonella* isolation from water samples; frequency of *Salmonella* detection was two-times greater in watersheds with poultry operations compared to watersheds without poultry operations. Overall, our findings suggest that one approach to managing the food safety risks associated with *Salmonella* contamination of preharvest surface water in NY is to address upstream sources of human and poultry fecal contamination. Such efforts could include identifying and addressing failing infrastructure upstream of irrigation pumps or treating irrigation water sources downstream of campgrounds, poultry farms or other fecal sources.

Our failure to find evidence of an association between *L. monocytogenes* and fecal indicator bacteria (FIBs, e.g., *E. coli*) is not unexpected as past studies also failed to find positive associations between *L. monocytogenes* and FIBs in water (e.g.,Schaffter et al., 2004; Wilkes et al., 2009; Economou et al., 2013; Weller et al., 2020). For instance, in their survey of the microbial quality of the South Nation River Basin, Wilkes et al. (2009) found a negative association between the likelihood of *L. monocytogenes* isolation and *E. coli* levels. While expected, our failure to identify an association between *L. monocytogenes* isolation and fecal indicators is interesting given the identification of positive associations between *L. monocytogenes* isolation and sources of human (e.g., campgrounds, wastewater discharge sites) and livestock (e.g., dairy farms) fecal contamination in this and other studies (Watkins and Sleath, 1981; Dijkstra, 1982; Paillard et al., 2005; Lyautey et al., 2007; Odjadjare et al., 2010; Strawn et al., 2013a; Weller et al., 2016). The failure to identify an association between *L. monocytogenes* and fecal indicators but the identification of an association between *L. monocytogenes* and sources of fecal contamination may be due to the fact *L. monocytogenes* is a microbe adapted to living in non-host environments (Vivant et al., 2013) but the FIBs used here are not (Lee et al., 2008; Bae and Wuertz, 2009). Thus, even if *L. monocytogenes* contamination is initially of fecal origin, *L. monocytogenes* may not correlate to presence or levels of fecal indicators since *L. monocytogenes* can persist in non-host environments longer than the indicators. Overall, these findings are illustrative of the need for alternatives to traditional indicator-based water quality tests for identifying when and where *L. monocytogenes* contamination of agricultural water sources is likely to occur.

### Rain Events, Ditches, and Other Factors Associated With Increased Stream Inflows Were Positively Associated With Pathogen Detection and Appeared to Mediate the Relationship Between Land Use and *L. monocytogenes* Detection

Multiple factors included in the analyses conducted here were hydrological in nature (e.g., flow rate), or influenced stream hydrology and inflows (e.g., presence of upstream ditches, rainfall). Interestingly, we found evidence of associations between these “hydrological” factors and detection of all targets in the present study. For example, the odds of *eaeA-stx* codetection was approx. two times greater when a ditch intersected the stream channel near the sample site compared to when no ditch was present. Similarly, we found evidence that increased rainfall was associated with an enhanced risk of *Salmonella*, *Listeria* spp. (excluding *monocytogenes*), and *L. monocytogenes* detection. Overall, these findings are not unexpected since past studies have found associations between “hydrological” factors and pathogen isolation (e.g., Goyal et al., 1977; Baudart et al., 2000; Kistemann et al., 2002; Cooley et al., 2007; Haley et al., 2009; Wilkes et al., 2009, 2011; Walters et al., 2011; Luo et al., 2015; Harris et al., 2018). A study that examined associations between environmental factors and pathogen isolation from Ontario river water only detected *Salmonella* when surface water discharge rates were elevated, which led the authors to conclude that events that promote off-farm and in-stream transfer of microbes (e.g., rain events) must occur for detection of *Salmonella* in the sampled rivers (Wilkes et al., 2011). Overall, the association between hydrological factors and microbial target detection is logical since hydrological factors are associated with processes that facilitate pathogen movement from terrestrial to freshwater environments. Falbo et al. (2013) investigated the role of ditches in bacterial loading of central New York waterways and found that, on average, 22% of a watershed’s area drains to roadside ditches, which capture and transport runoff and associated-bacteria to waterways from road surfaces and adjacent land areas. Falbo et al. (2013) also found that *E. coll* was capable of surviving in ditch sediments during dry periods and of re-entering the water column following resuspension of these sediments during rain events, suggesting ditches are a source of bacterial contaminants as well as a conduit for bacterial movement. Thus, efforts focused on reducing pathogen movement and survival in ditches may provide one set of strategies for preventing foodborne pathogens from entering agricultural water sources. Rain events can also physically transport pathogens from terrestrial sources to freshwater systems through overland run-off and facilitate the release of pathogens from soil, feces and other matrices so pathogens are available for transport from terrestrial sources to freshwater systems (Thelin and Gifford, 1983; Muirhead et al., 2004, 2005; Guber et al., 2006, 2007, 2015; Boyer, 2008). The key role of rain (and associated increases in run-off and instream flow) in bacterial release and transport is evidenced by the repeated inclusion of rainfall as a model parameter (Guber et al., 2009, 2011, 2013) or an experimental condition in studies investigating bacterial release from terrestrial sources and transport to freshwater systems (Thelin and Gifford, 1983; Muirhead et al., 2004, 2005; Guber et al., 2006, 2007, 2015; Boyer, 2008). In the *L. monocytogenes* CTree reported here, rain and hydrological factors appear to mediate the relationship between land use and *L. monocytogenes* isolation. The *L. monocytogenes* CTree implies that, for the sampled streams, a high upstream dairy density was associated with an increased likelihood of *L. monocytogenes* isolation only when there was a recent rain event. While these findings may seem obvious, they suggest several strategies for managing food safety hazards in agricultural water. For instance, if potential sources of fecal contamination (e.g., dairy farms) or features that facilitate pathogen movement from terrestrial sources to agricultural water sources (e.g., ditches, culverts) are present upstream, growers may want to treat water from these sources prior to use or use an alternative water source following rain events. As suggested by past studies (e.g., Diaz et al., 2010, 2012), impeding overland flow from potential sources of pathogen contamination to agricultural waterways (e.g., by constructing wetlands and vegetated buffers, changing ditch hydrology to facilitate settling out bacteria) may help mitigate the food safety risks associated with preharvest water use. However, additional research is needed to (i) determine how such strategies could be incorporated into a larger comanagement framework, and (ii) experimentally quantify the risk reduction (if any) associated with implementations of such measures.

### Weather and Physicochemical Water Quality Appear to Be Key Factors Associated With *eaeA-stx* and *Salmonella* Detection, Respectively

The majority of splits in the *Salmonella* CTree were based on physicochemical water quality; water quality factors were also associated with the greatest change in odds of *Salmonella* isolation according to regression analysis. Similarly, air temperature comprised the competitor and surrogate splits in the *eaeA-stx* CTree, while flow rate (a proxy for unmeasured upstream rainfall) was more strongly associated with the likelihood of *eaeA-stx* codetection than any other factor except detection of ruminant FST markers according to regression analysis. These findings suggest that physicochemical water quality and weather are key factors associated with *Salmonella* isolation and *eaeA-stx* detection, respectively. While past studies also found significant associations between temperature and pathogenic *E. coll* detection, the direction of these associations varied between studies (Wilkes et al., 2011; Francy et al., 2013; Luo et al., 2015; Bradshaw et al., 2016; Truchado et al., 2018; Weller et al., 2020). For example, Wilkes et al. (2011) found that temperatures above 14 C were associated with a reduced likelihood of isolating *E. coli* O157:H7 from water samples collected in eastern Ontario, while the present study found a strong positive association between *eaeA-stx* detection and temperature. Moreover, a 2017 study that characterized factors associated with temporal variability in the microbial quality of New York streams and Arizona canals, reported a polynomial relationship between *eaeA-stx* detection and air temperature in both states (Weller et al., 2020). Indeed, the existence of a complex (e.g., polynomial) relationship between temperature and *eaeA-stx* codetection could explain why we found evidence of a significant association between temperature and eaeA-stx codetection using CTree but not regression analysis. While the nature of the temperature-pathogenic *E. coli* relationship varies in direction and shape between studies, the relationships between *Salmonella* isolation and dissolved oxygen (Liang et al., 2015; Bradshaw et al., 2016; Weller et al., 2020), pH (Liang et al., 2015; Bradshaw et al., 2016; Weller et al., 2020), and turbidity (Liang et al., 2015; Bradshaw et al., 2016; Partyka et al., 2018; Weller et al., 2020) appear to be consistent across studies. As part of their study on the association between fecal indicators and human pathogens in tropical surface waters, Liang et al. (2015) reported a positive correlation between *Salmonella* levels and turbidity. Similarly, the 2017 study discussed above identified a strong, positive monotonic relationship between *Salmonella* detection and turbidity in New York and Arizona, and a strong, negative monotonic relationship between *Salmonella* detection and dissolved oxygen levels in Arizona (Weller et al., 2020). Overall the findings of this and other studies (Liang et al., 2015; Bradshaw et al., 2016; Partyka et al., 2018; Weller et al., 2020) suggest that physicochemical water quality factors could be incorporated as supplementary indicators into traditional microbial indicator-based water quality monitoring programs, as suggested by past studies (Weller et al., 2020). For example, growers concerned about *Salmonella* may want to measure pH or turbidity levels in agricultural water sources immediately prior to use; if pH is low or turbidity is high, the grower may want to use an alternative source or treat the water. Integration of physicochemical water quality parameters into water testing would provide information growers can use to guide decision-making in real-time; however additional research is needed to clarify how exactly physicochemical water quality data can be integrated with traditional microbial testing to inform hazard management efforts.

## CONCLUSION

Due to the number of streams sampled (*N* = 68), this study is one of the most geographically widespread surveys of the food safety hazards present in New York agricultural water to-date, and therefore provides key insights into pathogen dynamics in New York freshwater environments. Using a diversity of data types and advanced geographic analyses this study identified combinations of environmental conditions (e.g., rain events were associated with *L. monocytogenes* isolation from samples collected downstream of dairy farms) associated with an increased likelihood of pathogen presence in agricultural water. As such, this study provides data that will enable growers to better manage the food safety risks associated with preharvest surface water use. Specifically, the associations identified here can help New York growers qualitatively assess the food safety hazards associated with preharvest water use for individual water sources and in real-time. For example, we found evidence that likelihood of *Salmonella* isolation was positively associated with the sources of human fecal contamination, and with physicochemical water quality. These findings suggest several hazard management strategies, including identifying and addressing failing infrastructure upstream of irrigation pumps or incorporating physicochemical water quality factors as supplementary indicators into traditional microbial indicator-based water quality monitoring programs. Our study also highlights the potential for unintended consequences when food safety management practices are implemented with a single aim in mind. For example, although we found evidence that *eaeA-stx* codetection was associated with forest-wetland cover, our findings also suggest conversion of forest-wetland cover to developed or agricultural land cover could increase the potential for *Salmonella* or *L. monocytogenes* contamination. Given the strong association between hydrological factors and detection of all microbial targets in the present study, strategies for reducing overland flow from potential sources of fecal contamination (e.g., dairy farms) to agricultural water sources may help mitigate bacterial contamination of agricultural water sources as suggested in previous studies (Díaz et al., 2010, 2012). However, additional research is needed to determine (i) how such strategies could be incorporated into a larger comanagement framework, and (ii) if implementation of these strategies could have unexpected, detrimental impacts on food safety and/or environmental health as suggested above. Overall, this is a hypothesis-generating study that can guide future research. For example, the associations identified here (e.g., between *Salmonella* and physicochemical water quality) can be used to develop models to predict when and where a specific water source has an enhanced risk of being contaminated by a given pathogen. Our findings can also be used to guide efforts to quantify trade-offs associated with implementing specific food safety practices on-farm (e.g., removing forest-wetland cover to reduce risks associated with pathogenic *E. coli* contamination). Moreover, the methods used to obtain (e.g., scraping Google, government permits) and analyze (e.g., flow path distance as opposed to Euclidean) the geospatial data in this study, are novel and can serve as a template for the integration of these data types into future studies, including in other produce-growing regions.

## Supplementary Material

Supplemental Material

## Figures and Tables

**TABLE 1 |:** FST qPCR assays used and their performance parameters.

Assay name	Target	References	F and R primer conc. (μM)	Probe conc. (μM)	*R*^2^	Eff. (%)^a^	LLOQ^b^
DG3	Canine	(Green et al., 2014b)	1.4	0.1	0.994	97.2	10
HF183	Human	(Bernhard and Field, 2000; Green et al., 2014a)	1	0.08	0.994	98.8	10
Modified GFD	Avian	(Green et al., 2012)	1	0.1	0.993	97.4	10
Rum2Bac	Ruminant	(Mieszkin et al., 2010)	0.3	0.1	0.991	99.9	10
CG4	*C. elegans* internal control	(Kirtane et al., 2019)	1.4	0.1	0.943	99.9	10

a Calculated using %Eff = (10^(−1/slope)^)-1, where slope is the assay-specific slope of 4–6 pooled standard curves constructed using synthetic gBlocks® (IDT).

b Assay lower limits of quantification (gBlock® copies/reaction).

**TABLE 2 |:** Number of times each target was detected in a given watershed.

No. of Times Target Detected	Culture-Confirmed						PCR-Screen Positive^d^	
	*Listeria*^a^					*Salmonella*^c^		
	*Listeria* spp. excluding *monocytogenes*^b^				*monocytogenes*		*eaeA*	*stx*
	*innocua*	*marthii*	*seeligeri*	*welshimeri*				

**IN STREAMS SAMPLED TWICE (*N* = 8)**								

Zero	8	8	6	6	5	1	0	0
Once	0	0	1	2	3	1	0	4
Twice	0	0	1	0	0	3	8	4

**IN STREAMS SAMPLED THRICE (*N* = 60)**								
Zero	49	55	43	47	44	13	0	3
Once	10	5	16	11	15	30	0	12
Twice	1	0	1	1	1	12	6	26
Thrice	0	0	0	1	0	5	54	19

a Overall L. innocua, L. marthii, L. monocytogenes, L. seeligeri, and L. welshimeri were isolated from 6% (12/196), 3% (5/196), 10% (20/196), 11% (21/196), and 9% (18/196) of samples, respectively. One sample was positive for both L. monocytogenes and L. marthii, and one sample was positive for both L. innocua and L. seeligeri. L. innocua, L. marthii, L. monocytogenes, L. seeligeri, and L. welshimeri were isolated from 16% (11/68), 7% (5/68), 28% (19/68), 28% (19/68), and 22% (15/68) of streams, respectively.

b Listeria spp. excluding monocytogenes was isolated from 28% (55/196) of samples and 71% (48/68) of streams.

c Salmonella was isolated from 42% of samples (79/196) and 75% (51/68) of streams. Four samples were PCR-screen positive for Salmonella but culture-negative for Salmonella; these samples were not considered positive for Salmonella in the analyses reported here.

d The eaeA and stx genes were detected in 96% (190/196) and 68% (133/196) of samples, respectively. Additionally, the eaeA and stx genes were detected in 100% (68/68) and 96% (65/68) of streams, respectively. All stx-positive samples were also eaeA-positive.

**TABLE 3 |:** Ability of modified Moore swabs (mMS) compared to 0.45 um filters to recover *Listeria* from 29 paired 9-L and 1-L grab samples, respectively.

Species^a^	Prevalence (No. Pos.)		Proportion of		*P-value* for McNemar’s χ^2b^	Kappa^c^	
	mMS	Filters	Pos. Agreement	Neg. Agreement		Score (95% CI)	*P-value*
*L. seeligeri*	7% (2)	17% (5)	29%	90%	0.375	0.21 (−0.42, 0.84)	0.278
*L. monocytogenes*	3% (1)	24% (7)	0%	84%	0.070	−0.06 (−0.69, 0.56)	0.581
*Listeria* spp. excluding *monocytogenes*	7% (2)	38% (11)	15%	76%	0.012	–	–
*Listeria* spp. including *monocytogenes*	10% (3)	62% (18)	10%	49%	<0.001	–	–

a Due to the small number of samples that were L. innocua, L. marthii, and L. welshimeri positive (i.e., two samples each) analyses could not be performed to compare recovery using mMS and 0.45 μm filters.

b McNemar’s χ^2^ square tests for symmetry in a two-by-two contingency table; a P < 0.05 indicates significant disagreement in the number of mMS and the number of filters that were positive for the target organism.

c Cohen’s Kappa score is a measure of the level of agreement between two tests beyond what would have been expected by random chance. A p < 0.05 indicates that the extent of agreement is greater than expected by random chance. Due to the identification of significant disagreement in Listeria spp. recovery by mMS compared to 0.45 um filter by McNemar’s χ^2^ test, the kappa test was not performed to determine if there was significant agreement in Listeria spp. recovery by mMS compared to 0.45 μm filter.

**TABLE 4 |:** Associations between detection of each microbial or molecular target, and weather and water quality factors according to generalized linear mixed models^a^.

Outcome	Factor	Change in odds	95% CI ^b^	*P*-value
*eaeA-stx* Codetection				
	Avg. Solar Radiation 0-1d BSC^c^ (MJ/m^2^)	0.37	0.12, 4.15	0.084
	*E. coli* Levels (log_10_ MPN/100-mL)	1.77	0.98, 3.20	0.057
	Flow Rate (m/s)	2.90	0.86, 9.77	0.087
	Rum2Bac, Concentration (log_10_ Copies/100-mL)	1.64		0.012
	Rum2Bac, Detection (Failure to Detect = Reference)	4.25	1.35, 13.37	0.013
*Listeria* species excluding *L. monocytogenes*				
	Avg. Solar Radiation 2–3 d BSC (MJ/m^2^)	0.16	0.02, 1.29	0.085
	Avg. Air Temperature 20–30 d BSC (C)	0.93	0.86, 1.00	0.061
	Dissolved Oxygen Levels (mg/L)	1.34	1.06, 1.69	0.015
	*E. coli* Levels (log_10_ MPN/100-mL)	0.50	0.26, 0.96	0.036
	PH	3.65	1.14, 11.64	0.029
	Total Rainfall 2–3 d BSC (cm)	3.28	1.44, 7.51	0.005
	Total Rainfall 10–20 d BSC (cm)	0.78	0.59, 1.03	0.081
	Turbidity (log NTUs)	0.36	0.14, 0.94	0.037
	Water Temperature (C)	0.92	0.84, 0.99	0.029
*L. monocytogenes* Isolation				
	Turbidity (log_10_ NTUs)	0.32	0.09, 1.08	0.066
*Salmonella* Isolation				
	Avg. Solar Radiation 0–1 d BSC (MJ/m^2^)	0.27	0.09, 0.77	0.015
	Avg. Solar Radiation 2–3 d BSC (MJ/m^2^)	0.36	0.12, 1.08	0.068
	Avg. Air Temperature 10–20 d BSC (C)	0.94	0.87, 1.01	0.095
	Dissolved Oxygen Levels (mg/L)	0.74	0.72, 0.89	0.001
	*E. coli* Levels (log_10_ MPN/100-mL)	1.83	1.08, 3.09	0.025
	HF183, Concentration (log_10_ copies/100-mL)	1.24	1.01, 1.52	0.039
	HF183, Detection (Failure to Detect = Reference)	1.78	0.91, 3.50	0.095
	pH	0.24	0.10, 0.58	0.002
	Rainfall 0–1 d BSC (cm)	2.18	1.31, 3.62	0.003

a Since this was a hypothesis-generating study, two thresholds were used for interpreting the results of the GLMMs. Specifically, P< 0.05 indicated that likelihood of microbial target detection and the factor were significantly associated, while a 0.05≤P<0.10 indicated the presence of a potential relationship that warrants investigation in future studies.

b CI, Confidence interval.

c BSC, before sample collection.

**TABLE 5 |:** Associations between detection of each microbial or molecular target, and spatial factors according to generalized linear mixed models^a^.

Outcome	Factor	Change in Odds	95% CI^b^	*P*-value
*eaeA-stx* Codetection				
	Bottom Substrate, Cobble or Larger (Absent = Reference)	0.50	0.24, 1.05	0.068
	Developed Non-Open Space, IDW ^c^ % of Flood Plain	0.92	0.85, 0.99	0.012
	Developed Non-Open Space, IDW % of Stream Corridor	0.93	0.86, 1.00	0.036
	Developed Non-Open Space, IDW % of Total Watershed	0.95	0.90, 1.00	0.034
	Developed Open Space, IDW % of Flood Plain	0.95	0.91, 0.99	0.012
	Developed Open Space, IDW % of Stream Corridor	0.95	0.91, 0.99	0.010
	Developed Open Space, IDW % of Total Watershed	0.93	0.88, 0.99	0.018
	Ditch, Present Immediately Upstream of Site (Absent = Reference)	2.17	1.02, 4.62	0.044
	Forest Wetland, IDW % of Flood Plain	1.02	1.00, 1.04	0.022
	Forest Wetland, IDW % of Stream Corridor	1.02	1.00, 1.05	0.038
	Open Water, IDW % of Flood Plain	0.98	0.95, 1.00	0.043
	Open Water, IDW % of Stream Corridor	0.90	0.81, 0.99	0.033
	Open Water, IDW % of Total Watershed	0.83	0.69, 0.99	0.033
	Wastewater Discharge Sites, Upstream Density (per 10 km^2^)	0.23	0.04, 1.30	0.096
	In-stream Waterbodies, Flow Path Distance to Nearest (km)	1.10	1.01, 1.19	0.032
	In-stream Waterbodies, Present Upstream (Absent = Reference)	2.04	0.96, 4.35	0.065
*Listeria* species excluding *L. monocytogenes*				
	Bottom Substrate, Coarse Gravel (Absent = Reference)	2.29	0.97, 5.41	0.059
	Bottom Substrate, Fine Gravel (Absent = Reference)	2.12	0.87, 5.16	0.097
	Bottom Substrate, Organic Matter (Absent = Reference)	0.42	0.16, 1.10	0.077
	Bottom Substrate, Sand (Absent = Reference)	0.45	0.19, 1.04	0.062
	Campgrounds, Present Upstream (Absent = Reference)	0.37	0.13, 1.11	0.076
	Dairy Operations, Flow Path Distance to Nearest (km)	0.84	0.03. 0.73	0.027
	Dairy Operations, Present Upstream (Absent = Reference)	0.26	0.75, 0.98	0.028
	Ditch, Present Immediately Upstream of Site (Absent = Reference)	0.41	0.16, 1.05	0.064
	In-stream Waterbodies, Flow Path Distance to Nearest (km)	0.86	0.75, 0.98	0.028
	Livestock Operation, Present Upstream (Absent = Reference)	0.27	0.08, 0.93	0.039
	Pasture, IDW % of Flood Plain	1.03	1.00, 1.07	0.070
	Pig Farms, Present Upstream (Absent = Reference)	0.16	0.04, 0.69	0.014
	Pig Farms, Upstream Density (per 10 km^2^)	0.00^d^	0.00, 5.17	0.077
	Stables, Present Upstream (Absent = Reference)	0.32	1.06, 1.67	0.018
*L. monocytogenes* Isolation				
	Campgrounds, Upstream Density (per 10 km^2^)	565.4^e^	0.91, 3.50*10^5^	0.053
	Developed Non-Open Space, 0–100m Upstream of Site (%)	0.96	0.91, 1.01	0.086
*Salmonella* Isolation				
	Bottom Substrate, Cobble or Larger (Absent = Reference)	0.55	0.30, 1.01	0.054
	Campground, Flow Path Distance to Nearest (km)	0.94	0.88, 1.01	0.068
	Dairy Operations, Upstream Density (per 10 km^2^)	0.47	0.28, 0.81	0.007
	Pasture, 0–100m Upstream of Site (%)	0.99	0.97, 1.00	0.053
	Developed Open Space, IDW % of Stream Corridor	1.03	1.00, 1.06	0.096
	Ditch, Stormwater Outfalls Present Upstream (Absent = Reference)	2.04	1.03, 4.05	0.042
	Poultry Operations, Flow Path Distance to Nearest (km)	0.93	0.86, 1.00	0.060

a Since this was a hypothesis-generating study, two thresholds were used for interpreting the results of the GLMMs. Specifically, P< 0.05 indicated that likelihood of microbial target detection and the factor were significantly associated, while a 0.05 ≤P<0.10 indicated the presence of a potential relationship that warrants investigation in future studies.

b CI, Confidence interval.

c IDW, Inverse distance weighted.

d The odds ratio was >0.000 but <0.001.

e This confidence interval is wide due to sparse data bias, which is a product of the low prevalence of L. monocytogenes (10%; 20/196) and of watersheds with campgrounds upstream of the sampling site (22%; 16/71); 54% of L. monocytogenes-positive samples were collected from watersheds with campgrounds upstream.
