# Supplementary material for: Landscape, Water Quality, and Weather Factors Associated With an Increased Likelihood of Foodborne Pathogen Contamination of New York Streams Used to Source Water for Produce Production
Source: Front Sustain Food Syst. Author manuscript; Available in PMC 2020 May 21. (PMC7241490; doi:10.3389/fsufs.2019.00124)
Supplement: Supplemental Material [file NIHMS1583649-supplement-Supplemental_Material.docx]

Figure S1: Scatterplots showing the change in weather and physicochemical water quality parameters over the course of the study.


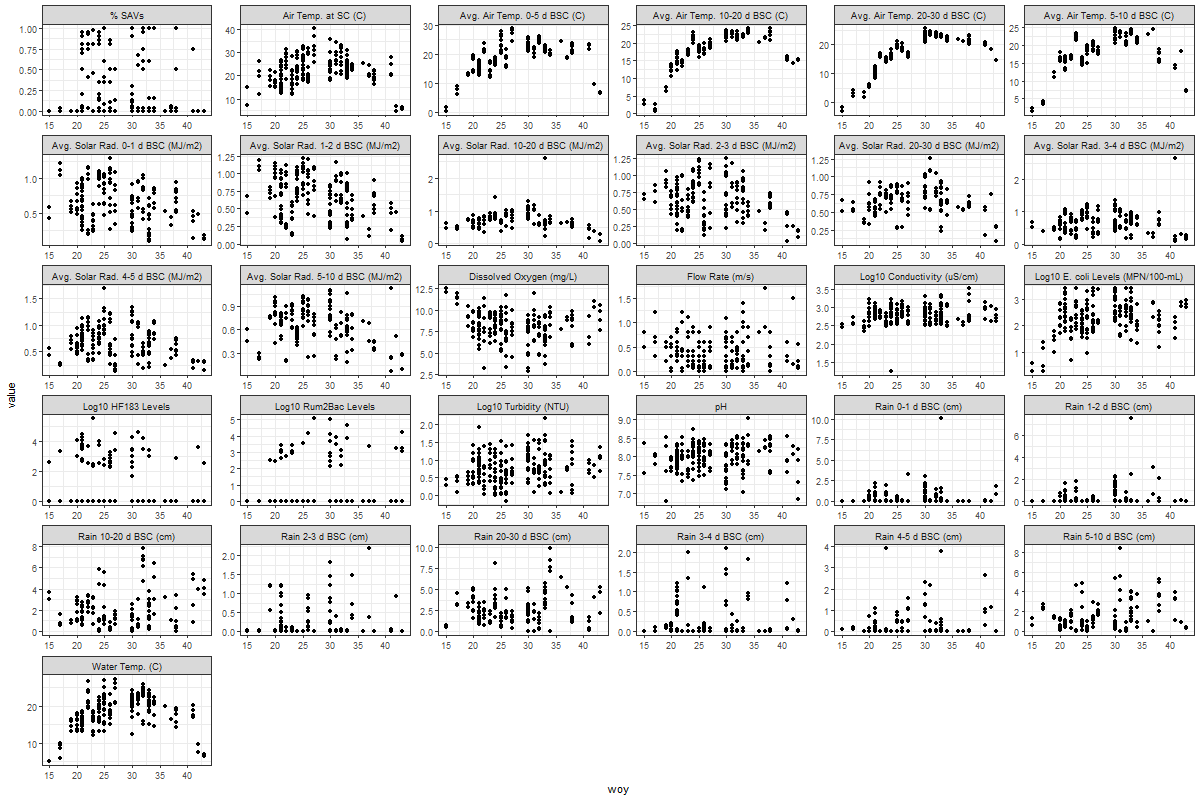


Figure S2: Matrix showing the correlation between weather and water quality factors in NY.


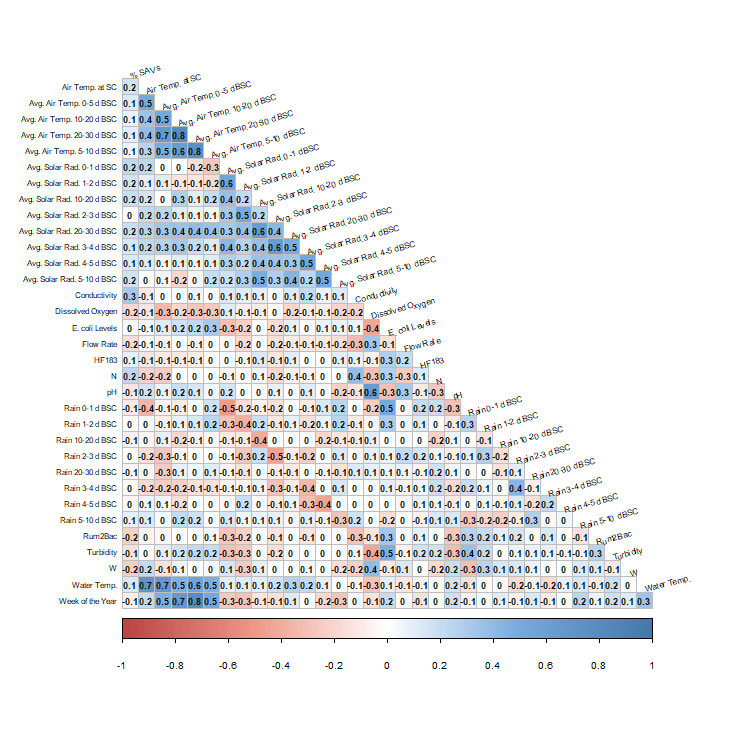


Figure S3: Matrix showing the correlation between spatial factors in NY (Deep Red = -1; Deep Blue =1; see the scale for Figure S4).


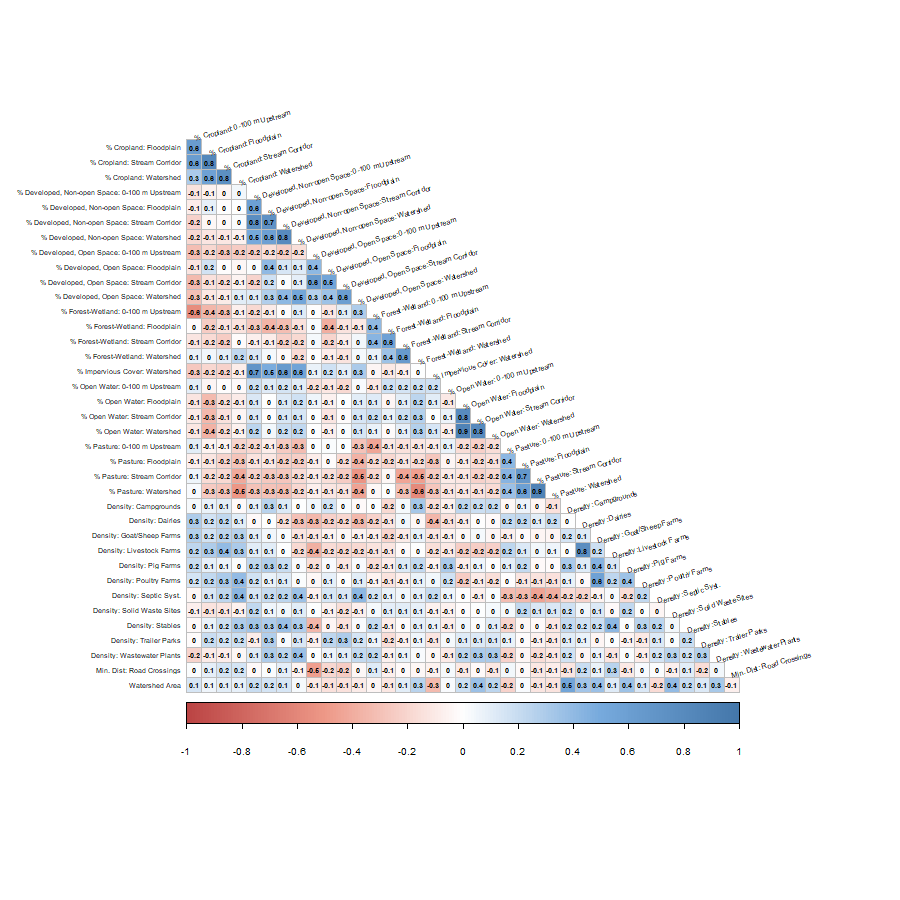


Table S1: Sensitivity and specificity of the modified GFD assay based on the analysis of fecal samples of known origin.

| Species | Common Name | Age (days) | Sample ID | ng/ul DNA^a^ | Copies/ng^b^ |
| --- | --- | --- | --- | --- | --- |
| *Aix sponsa* | Wood Duck | Unknown | WoDu 1SB | 1.08 | 12202.73 |
| *Aix sponsa* | Wood Duck | Unknown | WoDu 2SB | 1.42 | 6650.41 |
| *Branta bernicia* | Atlantic Brant | Unknown | ATBR 1FP | 31.2 | 2741.03 |
| *Branta bernicia* | Atlantic Brant | Unknown | ATBR 7FP | 7.1 | 5784.51 |
| *Branta bernicia* | Atlantic Brant | Unknown | ATBR 9FP | 1.69 | 11245.65 |
| *Branta canadensis* | Canada Goose | Unknown | CaGo 1BP | 14.6 | Below LOD |
| *Branta canadensis* | Canada Goose | Unknown | CaGo 1SB | 0.31 | 73.83 |
| *Branta canadensis* | Canada Goose | Unknown | CaGo 2BP | 4.02 | 3.18 |
| *Branta canadensis* | Canada Goose | Unknown | CaGo 3BP | 2.38 | 12.86 |
| *Branta canadensis* | Canada Goose | Unknown | CaGo 4BP | 1.86 | 4.29 |
| *Branta canadensis* | Canada Goose | Unknown | CaGo 5BP | 1.63 | 224.69 |
| *Laurus delawarensis* | Ring-billed Gull | Unknown | RBGU 1BP | 0.144 | 453.32 |
| *Laurus delawarensis* | Ring-billed Gull | Unknown | RBGU 2BP | 1.21 | Below LOD |
| *Laurus delawarensis* | Ring-billed Gull | Unknown | RBGU 3BP | 2.92 | 319894.35 |
| *Bos taurus* | Cow | 1643 | 284 | 84.4 | Below LOD |
| *Bos taurus* | Cow | 1635 | 289 | 25.6 | Below LOD |
| *Bos taurus* | Cow | 1498 | 428 | 26 | Below LOD |
| *Bos taurus* | Cow | 1446 | 484 | 86.4 | Below LOD |
| *Bos taurus* | Cow | 1355 | 561 | 108 | Below LOD |
| *Bos taurus* | Cow | 1216 | 674 | 108 | Below LOD |
| *Bos taurus* | Cow | 1055 | 843 | 86.8 | Below LOD |
| *Bos taurus* | Cow | 897 | 987 | 72.4 | Below LOD |
| *Bos taurus* | Cow | 892 | 995 | 34 | Below LOD |
| *Bos taurus* | Cow | 873 | 1016 | 55.4 | Below LOD |
| *Bos taurus* | Cow | 858 | 1031 | 67.2 | 0.1 |
| *Bos taurus* | Cow | 99 | 1828 | 62.6 | Below LOD |
| *Bos taurus* | Cow | 95 | 1831 | 112 | Below LOD |
| *Bos taurus* | Cow | 92 | 1833 | 0.796 | Below LOD |
| *Bos taurus* | Cow | 90 | 1838 | 92.4 | 0.49 |
| *Bos taurus* | Cow | 79 | 1848 | 85.4 | Below LOD |
| *Bos taurus* | Cow | 76 | 1851 | 120 | Below LOD |
| *Bos taurus* | Cow | 75 | 1854 | 67 | Below LOD |
| *Bos taurus* | Cow | 74 | 1855 | 85 | Below LOD |
| *Bos taurus* | Cow | 73 | 1856 | 57.6 | Below LOD |
| *Bos taurus* | Cow | 73 | 1858 | 106 | Below LOD |
| *Bos taurus* | Cow | 73 | 1859 | 81.8 | Below LOD |
| *Bos taurus* | Cow | 24 | 1903 | 99.4 | Below LOD |
| *Bos taurus* | Cow | 23 | 1904 | 25.6 | Below LOD |
| *Bos taurus* | Cow | 23 | 1905 | 116 | Below LOD |
| *Bos taurus* | Cow | 21 | 1907 | 114 | Below LOD |
| *Bos taurus* | Cow | 20 | 1908 | 116 | Below LOD |
| *Bos taurus* | Cow | 19 | 1909 | 66 | Below LOD |
| *Bos taurus* | Cow | 17 | 1912 | 53.4 | Below LOD |
| *Bos taurus* | Cow | 16 | 1913 | 96 | Below LOD |
| *Bos taurus* | Cow | 16 | 1914 | 96.8 | Below LOD |
| *Bos taurus* | Cow | 9 | 1918 | 96.4 | Below LOD |
| *Bos taurus* | Cow | 7 | 1920 | 65.2 | Below LOD |
| *Bos taurus* | Cow | 6 | 1921 | 33 | Below LOD |
| *Bos taurus* | Cow | 3040 | 9242 | 80.8 | Below LOD |
| *Bos taurus* | Cow | 2571 | 9593 | 98.8 | Below LOD |
| *Bos taurus* | Cow | 2176 | 9894 | 13.3 | Below LOD |

^a^ After final elution in 100 ul elution buffer using FastDNA Spin Kit for Soil (MPBio, Irvine, CA, US)

^b^ In undiluted eluate post qPCR analysis with GFD assay

Table S2: Factors included in the analyses reported here. Values for all weather factors with the exception of temperature were calculated for 0-1, 1-2, 2-3, 3-4, 4-5, 5-10, 10-20 and 20-30 d before sample collection (BSC). Values for temperature were calculated for 0-5, 5-10, 10-20 and 20-30 d BSC due to the strong correlation between temperature 0-1, 1-2, 2-3, 3-4 and 4-5 d BSC.

| Factor | | | Description | Date | Citations and Websites |
| --- | --- | --- | --- | --- | --- |
| Data Extracted from Publicly-Available Databases | | | |  |  |
|  | Campgrounds | | | 2018 | www.management-ware.com |
|  |  | Present | Were campgrounds present upstream? |  |  |
|  |  | Density | Upstream density (no. of per 10 km^2^) |  |  |
|  |  | Min. distance | If present, the flow path distance to nearest (km) |  |  |
|  | Culverts: Carrying a waterway under a state highway and have a span of 5 to 20 feet | | | 2014 | (1–3) |
|  |  | Present | Were culverts present upstream? |  |  |
|  |  | Min. distance | If was present, the flow path distance to nearest (km) |  |  |
|  | Dams | |  | 2018 | (4) |
|  |  | Present | Were dam(s) present upstream? |  |  |
|  |  | Min. distance | If present, the upstream flow path distance to nearest (km). |  |  |
|  | Livestock Operations: Dataset generated by joining all livestock datasets | | | 2018 | NA |
|  |  | Present | Were livestock operations present upstream? |  |  |
|  |  | Density | Upstream density (no. of per 10 km^2^) |  |  |
|  |  | Min. distance | If present, the flow path distance to nearest (km) |  |  |
|  | Dairies | | | 2017 | NA |
|  |  | Present | Were dairies present upstream? |  |  |
|  |  | Density | Upstream density (no. of per 10 km^2^) |  |  |
|  |  | Min. distance | If present, the flow path distance to nearest (km) |  |  |
|  | Goat or Sheep Farms | | | 2018 | [www.management-ware.com](http://www.management-ware.com) |
|  |  | Present | Were goat and/or sheep farms present upstream? |  |  |
|  |  | Density | Upstream density (no. of per 10 km^2^) |  |  |
|  |  | Min. distance | If present, the flow path distance to nearest (km) |  |  |
|  | Stables | |  | 2018 | [www.management-ware.com](http://www.management-ware.com) |
|  |  | Present | Were stables present upstream? |  |  |
|  |  | Density | Upstream density (no. of per 10 km^2^) |  |  |
|  |  | Min. distance | If present, the flow path distance to nearest (km) |  |  |
|  | Pig Farms | |  | 2018 | [www.management-ware.com](http://www.management-ware.com) |
|  |  | Present | Were pig farms present upstream? |  |  |
|  |  | Density | Upstream density (no. of per 10 km^2^) |  |  |
|  |  | Min. distance | If present, the flow path distance to nearest (km) |  |  |
|  | Poultry Farms | |  | 2018 | [www.management-ware.com](http://www.management-ware.com) |
|  |  | Present | Were poultry farms present upstream? |  |  |
|  |  | Density | Density of poultry farms upstream (no. per 10 km^2^). |  |  |
|  |  | Min. distance | If present, the flow path distance to nearest (km). |  |  |
|  | Road Crossing | | The flow path distance to nearest point upstream where a road crossed the stream (km). | 2015 | (5) |
|  | Outfalls: Municipal stormwater outfalls along or near highways (presence is an indicator of urbanization) | | | 2008 | (6–8) |
|  |  | Present | Were stormwater outfalls present upstream? |  |  |
|  |  | Min. distance | If present, the flow path distance to nearest (km). |  |  |
|  | Wastewater Discharge Sites: Based on permits issued under the NYS^a^ Pollutant Discharge Elimination System (SPDES) | | | 2018 | (9) |
|  |  | Present | Were wastewater discharge sites present upstream? |  |  |
|  |  | Density | Upstream density (no. of per 10 km^2^) |  |  |
|  |  | Min. distance | If present, the flow path distance to nearest (km) |  |  |
|  | In-stream Waterbodies: Bodies of water within the stream channel (e.g., mill ponds, impoundments, lakes) | | | 2017 | (10) |
|  |  | Present | Were waterbodies upstream? |  |  |
|  |  | Min. distance | If present, the flow path distance to nearest (km) |  |  |
|  | Septic System Density | | Upstream density (no. per 10 km^2^). | 2011 | (11) |
|  | Solid Waste Site: Based on permits issued by NYS that allow application and spreading of manure, human septage, food processing, or other waste | | | 2019 | (12) |
|  |  | Present | Were solid waste facilities present upstream? |  |  |
|  |  | Density | Upstream density (no. of per 10 km^2^) |  |  |
|  | Trailer Parks: Based on permits issued by NYS for active mobile home parks (i.e., operating with accommodations for the placement of ≥ 5 homes) | | | 2019 | (13) |
|  |  | Present | Were trailer parks present upstream? |  |  |
|  |  | Density | Upstream density (no. of per 10 km^2^) |  |  |
|  |  | Min. distance | If present, the flow path distance to nearest (km) |  |  |
|  | Land Cover ^b^ | | | 2016 | (14, 15) |
|  |  | Open Water | Class 11 in National Land Cover Database (NLCD) |  |  |
|  |  | Cropland | Cropland; Class 82 in NLCD |  |  |
|  |  | Pasture | Pasture; Class 81 in NLCD |  |  |
|  |  | Developed, Open Space | Developed with < 20% impervious cover; Class 21 in NLCD (e.g., parks, low density residential) |  |  |
|  |  | Developed, Non-open Space | Developed with > 20% impervious cover; Class 22-24 in NLCD (e.g., suburban, urban areas) |  |  |
|  |  | For-Wet | Natural cover; Classes 41-43, 51-52, 90, and 95 in NLCD (i.e., forest, shrubland or wetland) |  |  |
|  |  | Impervious | Percent of upstream watershed that was under impervious cover |  |  |
|  | Watershed Area | | Total area of upstream watershed (10-km^2^) | - | - |
| Water Quality and Hydrological Conditions at Time of Sample Collection | | | |  |  |
|  | *E. coli* | | Log_10_ *E. coli* concentration in the waterway (MPN/100 mL) | - | - |
|  | HF183 | | Microbial source tracking (MST) marker that indicates human fecal contamination | - | - |
|  | Levels | | Log_10_ concentration (Copies/100 mL) |  |  |
|  | Detection | | Detection or failure to detect marker in sample. |  |  |
|  | Rum2Bac | | MST marker that indicates ruminant fecal contamination | - | - |
|  | Levels | | Log_10_ concentration (Copies/100 mL) |  |  |
|  | Detection | | Detection or failure to detect marker in sample. |  |  |
|  | Conductivity | | Conductivity (Log_10_ uS/cm) | - | - |
|  | Dissolved oxygen | | Dissolved oxygen levels (mg/L) | - | - |
|  | Flow rate | | Flow rate measured 3-6” below the surface (m/s) | - | - |
|  | pH | | pH | - | - |
|  | Turbidity | | Turbidity (Log_10_ NTU) | - | - |
|  | Water Temp. | | Water temperature (°C) | - | - |
| Field-Collected Site Data | | | |  |  |
|  | Ditch | | Did a roadside ditch intersect the stream < 20 m upstream of the sample site? | ~~-~~ | ~~-~~ |
|  | Road Parallel | | Was there a road parallel to the stream < 20 m upstream of the sample site? | ~~-~~ | ~~-~~ |
|  | Bottom Substrate: Composition of the stream bottom in the reach 10 m upstream of the sample site. The different categories of substrate were boulder, bedrock, cobble or larger, coarse gravel, fine gravel, sand, clay and organic matter. | | | ~~-~~ | (16) |
|  |  | Rocky | Was the substrate that comprised the majority of the bottom rocky (bedrock, boulder, cobble, or gravel) or not rocky (sand, clay, or organic matter/silt)? | - | - |
|  |  | Predominant Substrate | What substrate comprised the majority of the bottom (cobble/boulder/bedrock, coarse gravel, fine gravel, sand/organic matter/clay)? | - | - |
|  |  | Sand | Was sand present along the stream bottom? |  |  |
|  |  | Clay | Was clay present along the stream bottom? |  |  |
|  |  | Organic Matter | Was organic matter present along the stream bottom? |  |  |
|  |  | Cobble or Larger | Were cobble, boulders or bedrock along the stream bottom? |  |  |
|  |  | Fine gravel | Was fine gravel present along the stream bottom? |  |  |
|  |  | Coarse gravel | Was coarse gravel present along the stream bottom? |  |  |
|  | Submerged Aquatic Vegetation (SAV) | | | - | - |
|  |  | Percent | The percent of the stream bottom 0-10 m upstream covered by SAVS | - | - |
|  |  | Present | Were SAVs present or absent? | - | - |
| Temporal | | |  |  |  |
|  | Week of the Year | | No. of weeks since the week containing Jan. 1^st^ | - | - |
| Weather | | |  |  |  |
|  | Air Temp. at site | | Air temperature measured at the sampling site at the time of sample collection (°C) | - | - |
|  | Avg. Air Temp. | | Average temperature (°C) either 0-5, 5-10, 10-20 or 20-30 d before sample collection | - | [newa.cornell.edu](http://newa.cornell.edu/) |
|  | Avg. Solar Radiation | | Average solar radiation (MJ/m^2^) either 0-1,1-2, 2-3, 3-4, 4-5, 5-10, 10-20 or 20-30 d before sample collection | - | [newa.cornell.edu](http://newa.cornell.edu/) |
|  | Total rainfall | | Total rainfall (cm) either 0-1,1-2, 2-3, 3-4, 4-5, 5-10, 10-20 or 20-30 d before sample collection | - | [newa.cornell.edu](http://newa.cornell.edu/) |

^a^ New York State = NYS

^b^ For each land cover class we calculated the proportion of (i) the total watershed area, (ii) the stream corridor (i.e., area 0-60 m from the stream corridor), (iii) the flood plain (based on shapefile downloaded from NYS Department of Environmental Conservation), and (iv) the area immediately upstream (0-100 m) of the sampling site.

^c^ Limit of detection = LOD; the upper limit of detection for the *E. coli* and total coliforms assay was 2,419.6 MPN/100-mL.

Table S3: Summary statistics for continuous spatial factors.

| Factor | | | No. of Streams with Feature Absent ^a^ | Min. | Max. | Mean | SD ^b^ | Median | Quartiles | |
| --- | --- | --- | --- | --- | --- | --- | --- | --- | --- | --- |
|  |  |  |  |  |  |  |  |  | 1^st^ | 3^rd^ |
|  | Campgrounds | |  |  |  |  |  |  |  |  |
|  |  | Density (No. per 10 km2) | 0 | 0.00 | 0.25 | 0.02 | 0.06 | 0.00 | 0.00 | 0.00 |
|  |  | Min. distance (km) | 55 | 0.81 | 35.83 | 13.15 | 10.02 | 11.71 | 6.27 | 15.31 |
|  | Culverts, Min. distance (km) | | 12 | 0.40 | 11.15 | 4.94 | 2.94 | 4.28 | 2.70 | 7.39 |
|  | Dams, Min. distance (km) | | 21 | 0.01 | 17.32 | 5.73 | 4.18 | 4.46 | 3.11 | 7.16 |
|  | Livestock Operations | |  |  |  |  |  |  |  |  |
|  |  | Density (No. per 10 km2) | 0 | 0.00 | 8.02 | 1.32 | 1.24 | 1.18 | 0.61 | 1.58 |
|  |  | Min. distance (km) | 7 | 0.02 | 20.51 | 4.43 | 3.96 | 3.49 | 1.89 | 5.98 |
|  | Dairies | |  |  |  |  |  |  |  |  |
|  |  | Density (No. per 10 km2) | 0 | 0.00 | 2.94 | 0.82 | 0.63 | 0.74 | 0.42 | 1.17 |
|  |  | Min. distance (km) | 8 | 0.02 | 20.51 | 5.38 | 4.23 | 4.28 | 2.52 | 6.35 |
|  | Goat and Sheep Farms | |  |  |  |  |  |  |  |  |
|  |  | Density (No. per 10 km2) | 0 | 0.00 | 0.86 | 0.03 | 0.12 | 0.00 | 0.00 | 0.00 |
|  |  | Min. distance (km) | 57 | 0.60 | 65.38 | 17.05 | 19.35 | 10.59 | 5.14 | 14.77 |
|  | Stables | |  |  |  |  |  |  |  |  |
|  |  | Density (No. per 10 km2) | 0 | 0.00 | 6.69 | 0.25 | 1.00 | 0.00 | 0.00 | 0.08 |
|  |  | Min. distance (km) | 47 | 0.18 | 31.52 | 8.57 | 8.18 | 5.73 | 2.97 | 10.01 |
|  | Pig Farms | |  |  |  |  |  |  |  |  |
|  |  | Density (No. per 10 km2) | 0 | 0.00 | 0.45 | 0.02 | 0.06 | 0.00 | 0.00 | 0.00 |
|  |  | Min. distance (km) | 59 | 0.60 | 43.57 | 13.19 | 13.81 | 7.30 | 5.60 | 14.50 |
|  | Poultry Farms | |  |  |  |  |  |  |  |  |
|  |  | Density (No. per 10 km2) | 0 | 0.00 | 0.56 | 0.06 | 0.12 | 0.00 | 0.00 | 0.04 |
|  |  | Min. distance (km) | 51 | 0.60 | 36.28 | 8.38 | 9.62 | 5.42 | 2.54 | 8.25 |
|  | Road Crossing, Min. distance (km) | | 0 | 0.00 | 4.23 | 0.52 | 0.80 | 0.11 | 0.01 | 0.89 |
|  | Stormwater Outfalls, Min. distance (km) | | 55 | 0.00 | 28.05 | 6.70 | 7.64 | 4.24 | 1.42 | 8.64 |
|  | SPDES Discharge Permits ^b^ | |  |  |  |  |  |  |  |  |
|  |  | Density (No. per 10 km2) | 0 | 0.00 | 1.27 | 0.11 | 0.22 | 0.00 | 0.00 | 0.14 |
|  |  | Min. distance (km) | 43 | 0.42 | 34.03 | 9.15 | 7.75 | 7.98 | 3.59 | 13.54 |
|  | In-stream Waterbodies, Min. distance (km) | | 21 | 0.00 | 25.50 | 6.86 | 5.80 | 4.98 | 3.06 | 9.44 |
|  | Septic System Density (No. per 10 km2) | | 0 | 59.50 | 781.00 | 135.18 | 91.08 | 113.00 | 92.95 | 147.50 |
|  | Solid Waste Application Sites, Density (No. per 10 km2) | | 0 | 0.00 | 0.57 | 0.02 | 0.08 | 0.00 | 0.00 | 0.00 |
|  | Trailer Parks | |  |  |  |  |  |  |  |  |
|  |  | Density (No. per 10 km2) | 0 | 0.00 | 2.35 | 0.23 | 0.35 | 0.10 | 0.00 | 0.34 |
|  |  | Min. distance (km) | 27 | 0.13 | 34.38 | 8.00 | 8.12 | 5.16 | 2.50 | 11.23 |
|  | Total Watershed Under (IDW %) | |  |  |  |  |  |  |  |  |
|  |  | Open Water | 0.00 | 12.57 | 0.73 | 1.80 | 0.19 | 0.08 | 0.45 | 0.00 |
|  |  | Cropland | 0 | 0.52 | 50.63 | 17.95 | 10.39 | 15.69 | 10.43 | 22.70 |
|  |  | Pasture | 0 | 0.28 | 79.84 | 28.70 | 18.73 | 25.76 | 14.58 | 41.76 |
|  |  | Developed, Non-Open Space | 0.00 | 40.88 | 4.22 | 6.74 | 2.49 | 1.03 | 4.55 | 0.29 |
|  |  | Developed, Open Space | 0.00 | 33.89 | 7.58 | 5.69 | 6.04 | 5.27 | 7.08 | 3.28 |
|  |  | Forest-Wetland | 0 | 11.48 | 72.57 | 40.16 | 13.92 | 37.45 | 31.61 | 49.73 |
|  |  | Impervious | 0 | 0.00 | 19.68 | 0.77 | 2.37 | 0.28 | 0.15 | 0.58 |
|  | Watershed 0-100 m Upstream of the Sampling Site Under (%) | |  |  |  |  |  |  |  |  |
|  |  | Open Water | 0 | 0.00 | 4.32 | 0.06 | 0.51 | 0.00 | 0.00 | 0.00 |
|  |  | Cropland | 0 | 0.00 | 98.06 | 17.41 | 23.32 | 9.09 | 0.00 | 25.24 |
|  |  | Pasture | 0 | 0.00 | 76.92 | 7.96 | 15.54 | 0.00 | 0.00 | 9.49 |
|  |  | Developed, Non-Open Space | 0 | 0.00 | 84.17 | 12.48 | 16.68 | 6.00 | 0.00 | 17.37 |
|  |  | Developed, Open Space | 0 | 0.00 | 48.43 | 22.15 | 12.45 | 22.22 | 13.92 | 31.71 |
|  |  | Forest-Wetland | 0 | 0.00 | 90.38 | 39.11 | 26.02 | 44.23 | 14.52 | 56.65 |
|  | Stream Corridor Under (IDW %) | |  |  |  |  |  |  |  |  |
|  |  | Open Water | 0 | 0.00 | 19.61 | 1.50 | 3.64 | 0.19 | 0.06 | 0.94 |
|  |  | Cropland | 0 | 0.40 | 43.39 | 13.21 | 9.90 | 10.49 | 6.14 | 17.58 |
|  |  | Pasture | 0 | 0.11 | 58.08 | 15.02 | 14.09 | 10.18 | 5.46 | 21.04 |
|  |  | Developed, Non-Open Space | 0 | 0.03 | 25.12 | 3.70 | 4.57 | 2.56 | 1.01 | 4.24 |
|  |  | Developed, Open Space | 0 | 2.03 | 52.30 | 8.49 | 8.79 | 6.49 | 4.56 | 8.31 |
|  |  | Forest-Wetland | 0 | 20.03 | 85.32 | 57.82 | 15.42 | 60.30 | 49.63 | 69.55 |
|  | Floodplain Under (IDW %) | |  |  |  |  |  |  |  |  |
|  |  | Open Water | 0 | 0.00 | 85.20 | 4.64 | 13.14 | 0.48 | 0.06 | 2.10 |
|  |  | Cropland | 0 | 0.25 | 51.65 | 10.92 | 11.28 | 7.05 | 3.12 | 14.85 |
|  |  | Pasture | 0 | 0.00 | 75.32 | 10.32 | 13.45 | 6.67 | 1.37 | 14.39 |
|  |  | Developed, Non-Open Space | 0 | 0.00 | 24.86 | 3.33 | 4.44 | 1.68 | 0.80 | 3.91 |
|  |  | Developed, Open Space | 0 | 0.00 | 54.74 | 6.46 | 8.29 | 4.13 | 2.28 | 7.06 |
|  |  | Forest-Wetland | 0 | 11.79 | 94.16 | 64.01 | 18.53 | 67.76 | 50.86 | 78.11 |
|  | Watershed Area (10 km^2^) | | 0 | 0.96 | 85.00 | 11.34 | 16.25 | 4.67 | 3.15 | 12.21 |

^a^ No. of streams (for spatial data) with missing data for this parameter. The number of streams with missing data represents the number of watersheds where the given point source was absent; the number of watersheds with the point source present equals 71-(No. of Samples Missing Data)

^b^ SD= Standard Deviation

Table S4: Summary statistics for continuous weather and water quality factors.

| Factor | | Missingness ^a^ | Min. | Max. | Mean | SD ^b^ | Median | Quartiles | |
| --- | --- | --- | --- | --- | --- | --- | --- | --- | --- |
|  |  |  |  |  |  |  |  | 1^st^ | 3^rd^ |
| Microbial | |  |  |  |  |  |  |  |  |
|  | *E. coli* Levels (MPN/100-mL) | 2 | 2 | 2,512 | 214 | 4 | 209 | 91 | 537 |
|  | DG3 (Copies/100-mL) ^c^ | 0 | 0 | 76 | 0.4 | 5 | 0 | 0 | 0 |
|  | GFD (Copies/100-mL) ^e^ | 0 | 0 | 7,040 | 51 | 513 | 0 | 0 | 0 |
|  | HF183 (Copies/100-mL) ^e^ | 0 | 0 | 320,449 | 6 | 28 | 0 | 0 | 3 |
|  | Rum2Bac (Copies/100-mL) ^f^ | 0 | 0 | 117,490 | 4 | 19 | 0 | 0 | 0 |
| Field-Collected Data | |  |  |  |  |  |  |  |  |
|  | Log10 Conductivity (uS/cm) | 0 | 1.26 | 3.51 | 2.79 | 0.24 | 2.80 | 2.62 | 2.94 |
|  | Dissolved oxygen (mg/L) | 0 | 2.87 | 12.54 | 8.10 | 1.77 | 8.19 | 7.18 | 9.27 |
|  | Flow rate (m/s) | 13 | 0.00 | 1.70 | 0.41 | 0.34 | 0.30 | 0.15 | 0.60 |
|  | pH | 3 | 6.79 | 9.03 | 7.99 | 0.36 | 8.01 | 7.78 | 8.26 |
|  | Log10 Turbidity (NTUs) | 0 | -0.17 | 2.16 | 0.77 | 0.42 | 0.73 | 0.47 | 1.03 |
|  | Water temperature (C) | 0 | 4.95 | 27.30 | 18.40 | 4.43 | 18.75 | 16.00 | 21.23 |
|  | Submerged Aquatic Vegetation (% Stream Bottom Covered) | 40 | 0.00 | 1.00 | 0.26 | 0.36 | 0.02 | 0.00 | 0.50 |
| Weather | |  |  |  |  |  |  |  |  |
|  | Air Temp. at site (C) | 0 | 4.80 | 40.50 | 22.19 | 6.12 | 22.30 | 18.60 | 25.80 |
|  | Avg. Air Temp. (C) |  |  |  | - |  |  |  |  |
|  | 0-5 d BSC ^g^ | 0 | 0.65 | 28.94 | 19.43 | 5.10 | 20.44 | 16.63 | 22.82 |
|  | 5-10 d BSC | 0 | 1.60 | 24.77 | 18.47 | 4.84 | 19.06 | 15.60 | 22.73 |
|  | 10-20 d BSC | 0 | 0.73 | 24.28 | 18.41 | 4.78 | 19.35 | 15.87 | 22.06 |
|  | 20-30 d BSC | 0 | -2.87 | 25.68 | 17.32 | 6.20 | 18.98 | 14.77 | 22.49 |
|  | Avg. Solar Radiation (MJ/m2) |  |  |  |  |  |  |  |  |
|  | 0-1 d BSC | 0 | 0.09 | 1.29 | 0.66 | 0.30 | 0.63 | 0.44 | 0.93 |
|  | 1-2 d BSC | 0 | 0.04 | 1.22 | 0.69 | 0.29 | 0.71 | 0.44 | 0.93 |
|  | 2-3 d BSC | 0 | 0.03 | 1.25 | 0.65 | 0.28 | 0.64 | 0.43 | 0.86 |
|  | 3-4 d BSC | 0 | 0.09 | 2.64 | 0.68 | 0.32 | 0.68 | 0.44 | 0.93 |
|  | 4-5 d BSC | 0 | 0.14 | 1.69 | 0.71 | 0.29 | 0.70 | 0.49 | 0.91 |
|  | 5-10 d BSC | 0 | 0.07 | 1.12 | 0.66 | 0.22 | 0.69 | 0.52 | 0.80 |
|  | 10-20 d BSC | 0 | 0.08 | 2.60 | 0.72 | 0.28 | 0.71 | 0.60 | 0.83 |
|  | 20-30 d BSC | 0 | 0.09 | 1.26 | 0.67 | 0.18 | 0.68 | 0.57 | 0.77 |
|  | Total rainfall (cm) |  |  |  | - |  |  |  |  |
|  | 0-1 d BSC | 0 | 0.00 | 10.06 | 0.42 | 0.93 | 0.03 | 0.00 | 0.58 |
|  | 1-2 d BSC | 0 | 0.00 | 7.57 | 0.33 | 0.80 | 0.00 | 0.00 | 0.23 |
|  | 2-3 d BSC | 0 | 0.00 | 2.18 | 0.21 | 0.43 | 0.00 | 0.00 | 0.15 |
|  | 3-4 d BSC | 0 | 0.00 | 2.08 | 0.19 | 0.41 | 0.00 | 0.00 | 0.13 |
|  | 4-5 d BSC | 0 | 0.00 | 3.89 | 0.27 | 0.60 | 0.00 | 0.00 | 0.26 |
|  | 5-10 d BSC | 0 | 0.00 | 8.33 | 1.53 | 1.48 | 1.04 | 0.40 | 2.29 |
|  | 10-20 d BSC | 0 | 0.00 | 7.77 | 1.92 | 1.79 | 1.32 | 0.65 | 2.74 |
|  | 20-30 d BSC | 0 | 0.00 | 9.83 | 2.65 | 1.74 | 2.34 | 1.45 | 3.44 |

^a^ No. of samples with missing data for this parameter.

^b^ SD= Standard Deviation

^c^ Since only 1 of the 196 samples tested positive for the canine fecal source tracking (FST) marker DG3, DG3 detection was not included as a covariate in the regression or cTree analyses conducted here.

^d^ Since only 8 out of the 196 samples tested positive for the avian FST marker GFD, GFD detection was not included as a covariate in the regression or cTree analyses conducted here. For the 8 positive samples, the mean and median number of copies/100-mL were 1,251 and 467 (Min. = 64; Max. = 7,040; 1^st^ Quartile = 132; 3^rd^ Quartile =798).

^e^ Forty-nine out of 196 samples tested positive for the human FST marker HF 183. For the 49 positive samples, the mean and median number of copies/100-mL were 1,643 and 1,205 (Min. = 49; Max. = 320,449; 1^st^ Quartile = 353; 3^rd^ Quartile = 4,858).

^f^ Thirty-four out of 196 samples tested positive for the ruminant FST marker Rum2Bac. For the 34 positive samples, the mean and median number of copies/100-mL were 1,974 and 1,315 (Min. = 145; Max. = 117,490; 1^st^ Quartile = 610; 3^rd^ Quartile = 4,449).

^g^ BSC=before sample collection

Table S5: Percent variance in the likelihood of detecting each target that is accounted for (i) by the base model used in the generalized linear mixed models reported in Tables 3 and 4^a^, and (ii) when different spatial and temporal factors are included as random and fixed effects. The random effects models allow for comparison of the relative importance of each spatial or temporal factor on the likelihood of isolating the given target.

| Target | Random Effects^b^ |  | Fixed Effects | Pseudo R^2 d^ | |
| --- | --- | --- | --- | --- | --- |
|  |  |  |  | Marginal | Conditional |
| *eaeA*-*stx* | |  |  |  |  |
|  | County ^e^ |  | - | - | 0% |
|  | - |  | Latitude ^e^ | < 1% | - |
|  | - |  | Longitude ^e^ | 10% | - |
|  | Stream |  | - | - | 3% |
|  | Stream |  | Month | 94% | 95% |
|  | Stream |  | Week of Year | 17% | 24% |
|  | Stream |  | Day of the Week | 5% | 10% |
|  | Stream |  | Hour of the Day | 2% | 5% |
| *Listeria* spp. excluding *monocytogenes* | |  |  |  |  |
|  | County ^e^ |  | - | - | 12% |
|  | - |  | Latitude ^e^ | 6% | - |
|  | - |  | Longitude ^e^ | < 1% | - |
|  | Stream |  | - | - | 26% |
|  | Stream |  | Month | 4% | 25% |
|  | Stream |  | Week of Year | <1% | 26% |
|  | Stream |  | Day of the Week | 2% | 25% |
|  | Stream |  | Hour of the Day | < 1% | 26% |
| *L. monocytogenes* | County ^e^ |  | - | - | 6% |
|  | - |  | Latitude ^e^ | <1% | - |
|  | - |  | Longitude ^e^ | 1% | - |
|  | Stream |  | - | - | 0% |
|  | Stream |  | Month | 8% | 8% |
|  | Stream |  | Week of Year | 7% | 7% |
|  | Stream |  | Day of the Week | 6% | 6% |
|  | Stream |  | Hour of the Day | <1% | <1% |
| *Salmonella* |  |  |  |  |  |
|  | County ^e^ |  | - | - | 4% |
|  | - |  | Latitude ^e^ | 1% | - |
|  | - |  | Longitude ^e^ | 6% | - |
|  | Stream |  | - | - | 2% |
|  | Stream |  | Month | 73% | 75% |
|  | Stream |  | Week of Year | 2% | 4% |
|  | Stream |  | Day of the Week | 8% | 9% |
|  | Stream |  | Hour of the Day | 2% | 2% |

^a^ Based model includes week of the year as a fixed effect and stream as a random effect.

^b^ CI=Confidence Interval

^c^ Plot nested in field.

^d^ Marginal R^2^ is the variance explained by the fixed effects, while conditional R^2^ is the variance explained by the while model (fixed plus random effects).

^e^ Included as proxies for spatial factors that vary at a larger scale than the watershed-level (e.g., regional patterns).

***SALMONELLA* PRIMARY ENRICHMENT**

**Grow-Back For *Salmonella***

□ Take one 10 L sample.

□ Shake grab sample and filter it through a modified Moore swab.

□ Transfer the mMS to a sterile, pre-labeled Whirl-Pak bag.

□ Ethanol mMS cassette and allow to dry on a paper towel on the window sill away from samples. Rinse hose and set aside to be autoclaved.

□ Add 225 mL of buffered peptone water + novobiocin solution (BPW+N) to a Whirl-Pak. The concentration of novobiocin in the BPW+N is 20 mg/L.

□ Prepare positive control by inoculating 10 mL of BPW+N with 20 uL a 10^-4^ dilution of a suspension culture of FSL F6-0826 (ATCC 700408) in BPW+N.

□ Prepare negative control by adding 10 mL of BPW+N to sterile, Whirl-Pak sample bag.

□ Following incubation at 35 C for 22-26 h, transfer the sample to the cold room (4 C).

□ Store enrichments at 4C for further analysis

*FDA BAM permits holding primary enrichments (enrichment containing modified Moore swab) to be held at 4*°*C for up to 48 h..*

*********

**LYSATE PREPARATION**

**BEFORE STARTING ANYTHING ELSE**

□ Turn on the 37 C and 95 C heat blocks, and check to make sure cooling block is in a 4 C coold room.

□ Disinfect the capping/de-capping tools with 70% ETOH.

□ Check to make sure that for each sample there is a (i) BAX PCR tube, (ii) BAX PCR cluster tube, and (iii) sufficient lysis buffer, and protease.

**LYSATE PREPARATION**

□ Prepare lysis buffer by adding 150 uL of protease to 12 mL of buffer. Cap and invert 8-10 times. Place on ice.

NOTE: Smaller amounts of lysis reagent can be made by adding 12.5 uL protease to 1 mL lysis buffer.

□ Label lysis tubes.

□ Aliquot 200 uL of lysis buffer to each of the tubes.

□ Aliquot 5 uL of the enrichment into the lysis buffer. Do not run the sample down the side of the tube, add directly to the buffer.

□ Securely cap tubes as each strip is completed using the capping/de-capping tool. Disinfect capping/de-capping tool with 70% ETOH each time.

□ Confirm temperature of 37C heat block.

□ Place the prepared lysates on the 37C block and incubate for 20 min.

□ Confirm the temperature of the 95C heat block.

□ Transfer lysates from 37C block to 95C block and heat for 10 min.

□ Remove the lysates from the 95C block and place in cooling block that has been kept at 4C.

□ Remove the lysates from the 95C block and place in cooling block that has been kept at 4C for at least 5 minutes.

Place the lysates in a sample rack, date/initial the plate, and store the lysates at 4 C (≤1 week) or -20 C (≤ 2 weeks).

*********

**REAL-TIME BAX ASSAY**

□ Label tubes with sample numbers

□ Open rack file and sample plate map; make sure that the sample ID and BAX assay selected for each test is correct.

□ Launch BAX program

**Always do this step prior to preparing the samples, the instrument needs time to reach temperature**.

□ Remove appropriate number of sample strips from the kit and place them in the cooling block.

**Note: The strips containing the reagent pellet must be kept on the cold block and not held at room temperature for more than 30 minutes.**

□ Visually check to make sure each tube contains a white reagent pellet.

□ Disinfect capping/de-capping tools with 70% ETOH.

□ Carefully remove the caps using the de-capping tool. Discard caps.

**DO EACH STRIP ONE AT A TIME!!!!**

□ Hydrate the reagent pellet with 30 uL of lysate.

□ Disinfect capping/de-capping tools with 70% ETOH.

□ Re-cap the assay tubes with the optical caps using the capping tool.

**Note: Tubes must be resealed witin 10 minutes of being opened.**

□ For *Salmonella* **ONLY** hold samples for 10 minutes at 4 C before placing in instrument.

□ Place the samples on the instrument.

□ Select “Run Full Process”

**Don’t leave the instrument unattended until it shows that it is performing PCR.**

*********

**SELECTIVE ENRICHMENT OF BAX *SALMONELLA* POSITIVES SAMPLES**

□ Add 200 uL of I2-KI and 100 uL of Brillian Green to each TT tube.

□ Transfer 1 mL from the primary enrichment (i.e., enrichment in BPW+N) to 9 mL of TT broth.

□ Transfer 100 uL from the primary enrichment (i.e., enrichment in BPW+N) to 9.9 mL each of RV broth.

□ Incubate RV and TT in a shaking water bath for 24 at 42 C.

□ Streak 50 uL of each secondary enrichment on *Salmonella* CHROMagar plating media.

□ Streak 50 uL of each secondary enrichment on 50 uL on XLD.

□ Incubate the *Salmonella* CHROMagar plates at 37 C for 18-24 h.

□ Incubate the XLD plates at 35 C for 18-24 h.

*********

***invA* CONFIRMATION OF CULTURE-BASED PRESUMPTIVE *SALMONELLA* POSITIVE SAMPLES**

**If mauve colonies are present on the CHROMagar OR black colonies are present on the XLD:**

- *Salmonella* colonies appear mauve on *Salmonella* CHROMAgar and black on XLD. If mauve colonies are present on the CHROMagar or black colonies select up to four *Salmonella* colonies (i.e., mauve on CHROMagar or black on XLD; if possible select 2 colonies per media) to restreak for isolation onto BHI.
- Incubate BHI plates at 37 C for 18-24 h.
- Perform *invA* PCR using one colony from each of the BHI plates as described in Protocol [8.1.1.3.6](https://confluence.cornell.edu/download/attachments/146903797/8.1.1.3.6-InvA%20colony%20PCR%20for%20Salm%2008-09-16.pdf?version=1&modificationDate=1470774100000&api=v2) [[InvA colony PCR for Salmonella](https://confluence.cornell.edu/download/attachments/146903797/8.1.1.3.6-InvA%20colony%20PCR%20for%20Salm%2008-09-16.pdf?version=1&modificationDate=1470774100000&api=v2) **PCR Protocol]** on the Cornell Food Safety Wiki.

**If mauve colonies are NOT present on the CHROMagar AND black colonies are NOT present on the XLD or the PCR performed on the mauve/black colonies is negative**:

- Select up to 12 colonies. If possible select 6 colonies from the CHROMagar (preferentially select blue colonies as Arizonae and Diarizonae are know to appear blue on *Salmonella* CHROMagar) and 6 red (not yellow) colonies from XLD to restreak for isolation onto BHI. If multiple colony morphologies are present test representatives of each. Note media, and colony color in your lab notebook.
- Incubate the BHI plates at 37 C for 18-24 h.
- Perform *invA* PCR using one colony from each of the BHI plates as described in Protocol [8.1.1.3.6](https://confluence.cornell.edu/download/attachments/146903797/8.1.1.3.6-InvA%20colony%20PCR%20for%20Salm%2008-09-16.pdf?version=1&modificationDate=1470774100000&api=v2) [[InvA colony PCR for Salmonella](https://confluence.cornell.edu/download/attachments/146903797/8.1.1.3.6-InvA%20colony%20PCR%20for%20Salm%2008-09-16.pdf?version=1&modificationDate=1470774100000&api=v2) **PCR Protocol]** on the Cornell Food Safety Wiki.

**Pathogenic *E. coli* (*eaeA-stx* Detection)**

**PRIMARY ENRICHMENT**

**Goal (if performing enrichment outside of normal sample processing flow):**

**__________________________________________________________________________________________________**__________________________________________________________________________________________

**Primary Enrichment For STEC:**

□ Take one 10 L sample.

□ Shake grab sample and filter it through a modified Moore swab.

□ Transfer the mMS to a sterile, pre-labeled Whirl-Pak bag.

□ Ethanol mMS cassette and allow to dry on a paper towel on the window sill away from samples. Rinse hose and set aside to be autoclaved.

□ Add 225 mL of TSB supplemented with casamino acids to a final concentration of 10 g/L and with novobiocin to a final concentration of 8 mg/L (TSB+N).

□ Prepare positive control by inoculating 10 mL of TSB+N with 20 uL of a 10^-4^ dilution of a suspension culture of FSL F6-0699 in TSB+N.

□ Prepare negative control by adding 10 mL of TSB+N to sterile, Whirl-Pak sample bag.

□ Following incubation at 41 C for 15-24 h, transfer the sample to the cold room (4 C).

□ Store enrichments at 4C for further analysis

*FDA BAM permits holding primary enrichments (enrichment containing modified Moore swab) to be held at 4*°*C for up to 48 h..*

*********

**LYSATE PREPARATION**

**BEFORE STARTING ANYTHING ELSE**

□ Turn on the 37 C and 95 C heat blocks, and check to make sure cooling block is in a 4 C cold room.

□ Disinfect the capping/de-capping tools with 70% ETOH.

□ Check to make sure that for each sample there is a (i) BAX PCR tube, (ii) BAX PCR cluster tube, and (iii) sufficient lysis buffer and protease.

**LYSATE PREPARATION**

□ Prepare lysis buffer by adding 150 uL of protease to 12 mL of buffer. Cap and invert 8-10 times. Place on ice.

NOTE: Smaller amounts of lysis reagent can be made by adding 12.5 uL protease to 1 mL lysis buffer.

□ Label lysis tubes.

□ Aliquot 200 uL of lysis buffer to each of the tubes.

□ Aliquot 20 uL of the enrichment into the lysis buffer. Do not run the sample down the side of the tube, add directly to the buffer.

□ Securely cap tubes as each strip is completed using the capping/de-capping tool.

□ Confirm temperature of 37C heat block.

□ Place the prepared lysates on the 37C block and incubate for 20 min.

□ Confirm the temperature of the 95C heat block.

□ Transfer lysates from 37C block to 95C block and heat for 10 min.

□ Remove the lysates from the 95C block and place in cooling block that has been kept at 4C for at least 5 minutes.

Place the lysates in a sample rack, date/initial the plate, and store the lysates at 4 C (≤1 week) or -20 C (≤ 2 weeks).

*********

**REAL-TIME BAX ASSAY**

□ Label tubes with sample numbers

□ Open rack file and sample plate map; make sure that the sample ID and BAX assay selected for each test is correct.

□ Launch BAX program

**Always do this AT LEAST 30 min PRior to preparing the samples, the instrument needs time to reach temperature**.

□ Remove appropriate number of sample strips from the kit and place them in the cooling block.

**Note: The strips containing the PCR pellet must be kept on the cold block and not held at room temperature for more than 30 minutes.**

□ Visually check to make sure each tube contains a white reagent pellet.

□ Disinfect capping/de-capping tools with 70% ETOH.

□ Carefully remove the caps using the de-capping tool. Discard caps.

**DO EACH STRIP ONE AT A TIME!!!!**

□ Hydrate the reagent pellet with 30 uL of lysate.

□ Disinfect capping/de-capping tools with 70% ETOH and re-cap the assay tubes using the optical caps.

**Note: Tubes must be resealed witHin 10 minutes of being opened.**

□ Once the PCR tablets are fully dissolved place the samples on the instrument.

□ Select “Run Full Process”

**Don’t leave the instrument unattended until it shows that it is performing PCR.**

**Bibliography**

1. Bridge inventory manual. NY State Department of Transportation, Albany, NY. <https://www.dot.ny.gov/divisions/engineering/structures/repository/manuals/inventory/2006_nysdot_inventory_manual_r.pdf>. Accessed June 4, 2019.
2. Culvert inventory and inspection manual. NY State Department of Transportation, Albany, NY. <https://www.dot.ny.gov/divisions/engineering/structures/repository/manuals/inventory/2006_nysdot_inventory_manual_r.pdf>. Accessed June 4, 2019.
3. NYS ;arge culverts. NY State Department of Transportation, Albany, NY. <https://gis.ny.gov/gisdata/inventories/details.cfm?DSID=1255>. Accessed June 17, 2019.
4. Dams. NY State Department of Transportation, Albany, NY. <https://www.dot.ny.gov/divisions/operating/oom/transportation-maintenance/repository/CulvertInventoryInspectionManual.pdf>. Accessed June 4, 2019.
5. National transportation dataset. United States Geological Service Sioux Falls, SD. <https://catalog.data.gov/dataset/usgs-national-transportation-dataset-ntd-downloadable-data-collectionde7d2>. Accessed June 17, 2019.
6. Outfall and system mapping for illicit discharge detection and elimination (IDDE) in NY. NY State Department of Transportation, Albany, NY. ftp://ftp.dec.state.ny.us/dow/stormdocuments/ms4/illicit_discharge_detection_and_elimination/illicit_discharge_detection_and_elimination_assistance/guidance/IDDE NYS Mapping Doc FINAL 06 12 05 for FTP site.pdf. Accessed June 4, 2019.
7. Guidance on outfall mapping: What is an outfall, and what should be mapped. NY State Department of Transportation, Albany, NY. http://www.dec.state.ny.us/website/dow/MS4crit.pdf. Accessed June 4, 2019.
8. Methodology for the identification and survey of stormwater outfalls within designated MS4 locations for New York State. NY State Department of Transportation, Albany, NY. <http://www.dec.state.ny.us/website/dow/MS4crit.pdf>. Accessed June 4, 2019.
9. State pollutant discharge elimination system. NY State Department of Environmental Conservation, Albany, NY. <https://gis.ny.gov/gisdata/inventories/details.cfm?DSID=1010>. Accessed June 17, 2019.
10. National hydrography database. United States Geological Service, Sioux Falls, SD. <https://gis.ny.gov/gisdata/inventories/details.cfm?DSID=1010>. Accessed June 17, 2019.
11. Septic systems, New York State, 2011. New York Water Resources Insitute, Ithaca, NY. <https://cugir.library.cornell.edu/catalog/cugir-008164>. Accessed July 29, 2019.
12. Active solid waste management facilities. NY State Department of Environmental Conservation, Albany, NY. <https://data.ny.gov/Energy-Environment/Solid-Waste-Management-Facilities/2fni-raj8/data>. Accessed June 17, 2019.
13. Mobile home parks. NY State Department of Health, Albany, NY. <https://health.data.ny.gov/Health/Mobile-Home-Parks-Last-Inspection/d3mj-xg62>. Accessed June 17, 2019.
14. NLCD 2016 Land Cover (CONUS). United States Geological Service, Sioux Falls, SD. https://www.mrlc.gov/data?f%5B0%5D=category%3Aland cover. Accessed June 17, 2019.
15. NLCD 2016 Percent Developed Imperviousness. United States Geological Service, Sioux Falls, SD. https://www.mrlc.gov/data?f%5B0%5D=category%3Aurban imperviousness. Accessed June 17, 2019.
16. Unified stream assessment: a user’s manual. 2005. Center for Watershed Protection, Ellicott City, MD. <https://owl.cwp.org/mdocs-posts/urban-subwatershed-restoration-manual-series-manual-10/>.
